# Sodium–Glucose Cotransporter 2 Inhibitors in Aortic Stenosis: Toward a Comprehensive Cardiometabolic Approach

**DOI:** 10.3390/ijms26104494

**Published:** 2025-05-08

**Authors:** Paschalis Karakasis, Panagiotis Theofilis, Dimitrios Patoulias, Panayotis K. Vlachakis, Konstantinos Pamporis, Marios Sagris, Nikolaos Ktenopoulos, George Kassimis, Antonios P. Antoniadis, Nikolaos Fragakis

**Affiliations:** 1 Second Department of Cardiology, Hippokration General Hospital, Aristotle University of Thessaloniki, 54642 Thessaloniki, Greece; gksup@yahoo.gr (G.K.); aantoniadis@gmail.com (A.P.A.); fragakis.nikos@googlemail.com (N.F.); 2 First Cardiology Department, School of Medicine, Hippokration General Hospital, National and Kapodistrian University of Athens, 11527 Athens, Greece; panos.theofilis@hotmail.com (P.T.); vlachakispanag@gmail.com (P.K.V.); konstantinospab@gmail.com (K.P.); masagris1919@gmail.com (M.S.); nikosktenop@gmail.com (N.K.); 3 Second Propedeutic Department of Internal Medicine, Faculty of Medicine, School of Health Sciences Aristotle, University of Thessaloniki, 54642 Thessaloniki, Greece; dipatoulias@gmail.com

**Keywords:** aortic stenosis, SGLT2 inhibitors, heart failure, valve replacement, myocardial remodeling, fibrosis, inflammation, oxidative stress, cardiometabolic therapy

## Abstract

Aortic stenosis (AS), the most prevalent valvular heart disease, is increasingly recognized as an active disease process driven by a convergence of hemodynamic stress, inflammation, oxidative injury, and metabolic remodeling. While transcatheter and surgical valve replacement remain the standard interventions for severe AS, they fail to reverse the chronic myocardial remodeling that underlies adverse outcomes in many patients. Sodium–glucose cotransporter 2 (SGLT2) inhibitors have emerged as promising cardioprotective agents, with effects extending well beyond glycemic control. Recent mechanistic studies reveal that SGLT2 is expressed in the myocardium of patients with AS and is linked to pathways of fibrosis, inflammation, and energetic dysfunction. Experimental models and translational data demonstrate that SGLT2 inhibition attenuates maladaptive remodeling through modulation of TGF-β, NF-κB, NLRP3 inflammasome, and oxidative stress signaling while enhancing mitochondrial energetics and endothelial function. Importantly, clinical evidence from randomized and real-world studies suggests that SGLT2 inhibitors improve heart failure outcomes following valve replacement and may slow AS progression. This review integrates current pathophysiological insights with emerging molecular and clinical data to delineate the therapeutic rationale for SGLT2 inhibition in AS. By targeting both myocardial and valvular components of the disease, SGLT2 inhibitors may offer a novel disease-modifying strategy with potential implications across the AS continuum—from asymptomatic stages to the post-interventional setting. Ongoing and future trials are warranted to define optimal patient selection, timing, and biomarkers for response to SGLT2 inhibitor therapy in this increasingly high-risk population.

## 1. Introduction

Aortic valve stenosis (AS) is the most prevalent valvular heart disease in industrialized nations and a growing public health challenge in aging populations [1,2,3,4,5,6]. Characterized by progressive calcification and narrowing of the aortic valve, AS imposes chronic pressure overload on the left ventricle, triggering a cascade of maladaptive myocardial remodeling that includes hypertrophy, interstitial fibrosis, and, ultimately, heart failure [7,8,9]. Despite the widespread adoption of transcatheter and surgical aortic valve replacement (TAVI/AVR) as definitive interventions [10], clinical outcomes remain suboptimal in many patients, particularly those with advanced myocardial damage at the time of intervention [11,12,13,14,15,16,17,18]. Notably, structural remodeling often persists even after valve replacement, underscoring the need for adjunctive therapies capable of modifying the underlying pathophysiology [11,16,19].

Recent advances in cardiovascular pharmacotherapy have positioned sodium–glucose cotransporter 2 (SGLT2) inhibitors as promising agents in the management of heart failure [20,21,22]. Initially developed as glucose-lowering agents, SGLT2 inhibitors have demonstrated significant cardioprotective effects across diverse clinical settings and patient populations, including those without diabetes [23,24,25,26,27]. Emerging preclinical and clinical evidence suggests that these benefits may extend to patients with AS, where myocardial SGLT2 expression has been implicated in the pathogenesis of fibrosis, inflammation, oxidative stress, and metabolic dysfunction [28,29,30]. These findings raise the possibility that SGLT2 inhibitors may modulate key pathophysiological processes in AS and complement structural interventions such as TAVI or AVR.

This review explores the molecular and cellular mechanisms underlying AS progression, highlights the emerging role of SGLT2 inhibitors as modulators of cardiometabolic remodeling, and discusses the therapeutic implications of integrating SGLT2 inhibition into the management of aortic valve disease.

## 2. Pathophysiological Overview of Aortic Valve Stenosis

The literature search and selection process underlying this review are summarized in Supplementary Figure S1.

AS is increasingly recognized not as a passive degenerative condition but as an active, multifaceted disease driven by molecular, mechanical, inflammatory, and metabolic cues [31,32]. The pathological process evolves through two distinct yet overlapping phases: initiation and propagation, each contributing uniquely to the fibrotic and calcific degeneration of the aortic valve and subsequent myocardial remodeling [31,32].

### 2.1. Hemodynamic Stress and Pressure Overload

The hallmark of AS is the progressive narrowing of the aortic valve orifice due to fibrocalcific thickening of the valve cusps [33]. This structural abnormality imposes chronic pressure overload on the left ventricle (LV), necessitating increased myocardial wall tension to maintain cardiac output [34]. In the early stages, this pressure overload induces concentric hypertrophy of the LV myocardium—a compensatory response aimed at preserving systolic function [35]. However, over time, this adaptation becomes maladaptive, with impaired coronary perfusion, increased myocardial oxygen demand, and eventual LV decompensation [36,37,38]. As outlined by Shah et al. [31], maladaptive hypertrophy progresses to interstitial fibrosis, microvascular rarefaction, and systolic dysfunction, culminating in heart failure—a major determinant of post-intervention outcomes [39,40,41,42,43,44].

### 2.2. Myocardial Remodeling: Fibrosis, Microvascular Dysfunction, and Energetic Impairment

The sustained hemodynamic stress and neurohormonal activation in AS stimulate a cascade of profibrotic signaling pathways, including TGF-β, galectin-3, and matrix metalloproteinases, leading to collagen deposition and extracellular matrix expansion [45,46,47,48,49,50,51]. This structural remodeling stiffens the ventricular myocardium, impairing diastolic relaxation and coronary reserve [52,53,54]. Microvascular dysfunction—driven by increased wall stress, capillary rarefaction, and endothelial dysfunction—exacerbates ischemia and promotes further fibrosis. In advanced disease, impaired energetics [55], characterized by mitochondrial dysfunction and substrate switching from fatty acids to glucose, contribute to contractile inefficiency and myocardial vulnerability [56,57].

### 2.3. Inflammation, Oxidative Stress, and Metabolic Reprogramming in Valve and Myocardium

At the valvular level, disease initiation begins with endothelial injury due to shear stress [58], particularly on the fibrosa surface, triggering endothelial-to-mesenchymal transition (EndMT) [59] and infiltration of lipoproteins, such as Lp(a) and oxidized LDL [60,61,62]. These lipids provoke local inflammation, recruiting macrophages and T cells that release proinflammatory cytokines (IL-6, TNF-α, IL-1β) [63,64,65] and activate the NF-κB pathway [66]. Valvular interstitial cells (VICs) differentiate into myofibroblast- and osteoblast-like phenotypes, driving the development of fibrosis and calcification [47,67]. Apoptotic bodies and extracellular vesicles from VICs and immune cells serve as nucleation sites for hydroxyapatite deposition [68,69].

Metabolically, the AS myocardium undergoes significant reprogramming [70,71]. Downregulation of PPAR-α and mitochondrial enzymes, coupled with an increased reliance on glycolysis and ketone utilization, reflects a shift toward an energetically inefficient state [72,73]. These changes mirror those observed in pressure overload and heart failure, indicating a shared molecular substrate between valvular and myocardial disease components [71,74,75].

## 3. SGLT2 Inhibitors in Aortic Valve Stenosis: Mechanisms Beyond Glycemic Control

Given the complex interplay of hemodynamic stress, inflammation, oxidative damage, endothelial dysfunction, and metabolic dysregulation that underpins AS progression [76], therapeutic strategies that target these interconnected pathways are urgently needed [77]. Traditional interventions focus primarily on relieving valvular obstruction; yet, they leave the underlying myocardial and vascular pathology largely unaddressed. In this context, SGLT2 inhibitors have garnered considerable interest due to their ability to modulate multiple cellular and molecular processes implicated in both valvular and myocardial remodeling [78,79,80,81]. Although initially developed for glycemic control in diabetes, emerging evidence highlights their cardioprotective effects in non-diabetic settings—including AS—through mechanisms that extend well beyond glucose lowering (Table 1).

### 3.1. Molecular Targets in the AS Myocardium

**Figure 1:**
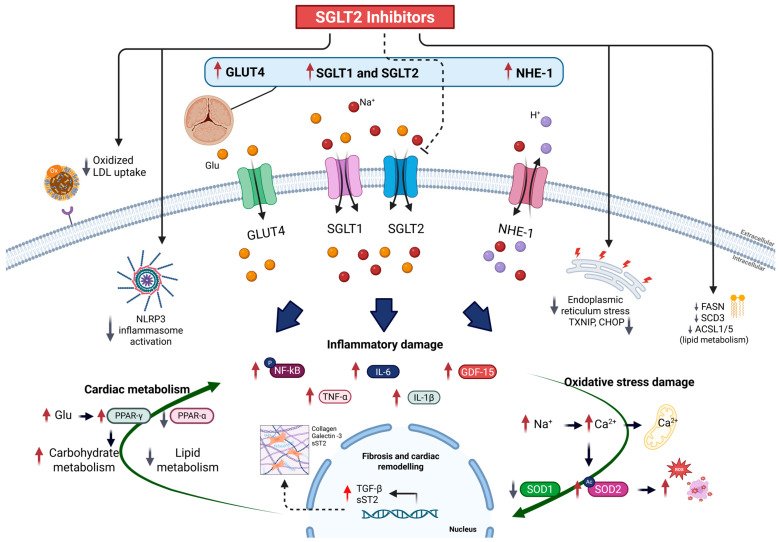
Mechanistic actions of SGLT2 inhibitors in aortic valve stenosis. SGLT2 inhibitors exert cardiometabolic benefits in aortic stenosis (AS) by modulating key molecular pathways involved in myocardial, vascular, and valvular remodeling. In the myocardium, SGLT2 inhibitors attenuate pressure overload-induced remodeling by suppressing profibrotic signaling (e.g., TGF-β, collagen I/III), reducing inflammatory activation (e.g., IL-6, NF-κB, NLRP3 inflammasome), and limiting oxidative stress through upregulation of antioxidant enzymes (e.g., SOD2, catalase, glutathione peroxidase). Metabolically, SGLT2 inhibitors restore myocardial energetic efficiency by enhancing mitochondrial ATP production, increasing ketone body utilization, and reducing cytosolic Na^+^/Ca^2+^ overload via NHE1 inhibition. These effects are supported by reactivation of PPAR-α signaling and attenuation of maladaptive PPAR-γ-dominant responses, promoting fatty acid oxidation over lipogenesis. Concurrently, lipotoxic enzyme expression (e.g., FASN, SCD3, ACSL1/5) is downregulated. In the vasculature, SGLT2 inhibitors preserve endothelial function by restoring eNOS activity, suppressing adhesion molecules (ICAM-1, VCAM-1), and reducing ER stress (↓ TXNIP, CHOP). Aortic stiffness is alleviated through improved vascular compliance and metabolic remodeling. At the valvular level, emerging evidence suggests SGLT2 inhibitors may inhibit oxLDL uptake, VIC osteogenic activation, and calcification. Together, these integrated effects support a disease-modifying role for SGLT2 inhibitors across the AS continuum. Solid lines with arrowheads indicate pathways or processes promoted by SGLT2 inhibitors; dashed lines with flat ends indicate inhibition.

SGLT2 expression, although traditionally thought to be restricted to the proximal renal tubules, has been identified in extra-renal tissues under pathological conditions, including the myocardium and vasculature [96,97]. Scisciola et al. [98] demonstrated significant myocardial SGLT2 expression in patients with severe AS, particularly those with low-flow, low-gradient phenotypes. Importantly, SGLT2 expression correlated positively with pro-fibrotic (TGF-\u03b2, collagen) and pro-inflammatory (IL-6, NF-\u03baB) markers while inversely correlating with antioxidant defenses such as SOD2 (Figure 1). This provides a rationale for the direct myocardial effects of SGLT2 inhibitors in AS [98].

Beyond SGLT2 itself, several key signaling nodes have been implicated in the SGLT2 inhibitors’ actions. The inhibition of the sodium–hydrogen exchanger 1 (NHE1) leads to reductions in intracellular sodium and calcium overload, thus alleviating mitochondrial dysfunction and contractile inefficiency. Alsereidi et al. [83] demonstrated that dapagliflozin modulates multiple inflammatory and oxidative pathways in cardiomyocytes, aortic endothelial cells (AECs), and stem cell-derived \u03b2-cells, including AKT/PI3K, NHE1, MAPK, and NLRP3 inflammasome suppression [83]. The antioxidant nuclear factor NRF2 was also modulated, indicating a broad cytoprotective profile [83].

### 3.2. Anti-Inflammatory and Antioxidative Effects

Chronic inflammation and oxidative stress are pivotal in the progression of AS, driving both valvular calcification and myocardial fibrosis. Multiple studies underscore the anti-inflammatory potential of SGLT2 inhibitors [99,100]. For example, Chandrasekar et al. [86] found that empagliflozin reversed oxidized LDL-induced suppression of RECK and inhibited MMP2/9 activation in aortic smooth muscle cells (SMCs) while suppressing NF-\u03baB signaling and pro-inflammatory cytokine release. Similarly, Sukhanov et al. [91] reported that empagliflozin blocked IL-17A-induced ROS production, NLRP3 inflammasome activation, and downstream release of IL-1\u03b2 and IL-18 in SMCs, independent of glucose levels.

In endothelial models, Campeau and Leask [87] demonstrated that empagliflozin attenuated tunicamycin-induced endoplasmic reticulum stress and NLRP3 activation via downregulation of TXNIP and CHOP, thereby preserving endothelial homeostasis. El-Daly et al. [93] further showed that empagliflozin improved NO-dependent vasodilation in hyperglycemia-exposed aortic rings by inhibiting oxidative signaling through NADPH oxidase and restoring eNOS activity.

Vascular effects have also been demonstrated in vivo. Liu et al. [50] reported that dapagliflozin reduced AAA formation by lowering MMP expression, reducing mural inflammation, and preserving SMC integrity. Ortega et al. [92] corroborated these findings, showing that empagliflozin inhibited Ang II-induced dissecting aneurysms in ApoE^−/−^ mice by targeting VEGF, MMPs, and MAPK/NF-\u03baB signaling.

Kawade et al. [88] extended these findings by demonstrating that luseogliflozin increased SOD2 expression and reversed free fatty acid-induced oxidative stress in the thoracic aorta of obese mice. These antioxidative effects likely contribute to the broader vasoprotective actions of SGLT2 inhibitors in AS.

### 3.3. Effects on Endothelial and Vascular Function

Endothelial dysfunction is a central feature of AS-associated vascular pathology. SGLT2 inhibitors have been shown to preserve endothelial integrity through several mechanisms. Ashry et al. [90] found that canagliflozin restored acetylcholine-induced aortic relaxation in hypercholesterolemic rabbits while reducing oxidative stress (MDA, NOx) and inflammation (CRP). In a pilot clinical study, Solini et al. [94] observed that dapagliflozin improved flow-mediated dilation, reduced aortic stiffness (PWV), and decreased renal resistive index within 48 h, independent of natriuresis or glycemic changes.

These vascular effects have been further corroborated in proteomic studies. Yue et al. [84] showed that empagliflozin downregulated fatty acid metabolic enzymes (FASN, SCD3, ACSL1, ACSL5) and improved aortic compliance in obese mice, indicating a direct role in modulating vascular lipid handling and stiffness.

### 3.4. Metabolic and Energetic Effects

As previously discussed, a defining feature of advanced AS is myocardial metabolic reprogramming, characterized by impaired fatty acid oxidation, dysfunctional mitochondrial respiration, and increased reliance on glycolysis and ketone bodies [70,71]. These metabolic shifts reduce ATP availability and impair cardiac efficiency, particularly under conditions of increased wall stress and restricted perfusion.

SGLT2 inhibitors have demonstrated the ability to promote a more favorable metabolic profile, restoring mitochondrial function and substrate flexibility [101]. In addition to enhancing ketone body handling, reducing cytosolic sodium and calcium overload, and increasing ATP generation [101,102,103], accumulating evidence supports a broader, more integrated role for these agents in reprogramming cellular energetics. Specifically, SGLT2 inhibitors appear to induce a starvation-mimetic state that activates evolutionarily conserved nutrient-sensing pathways, including AMPK [104], sirtuins (SIRT1/3/6) [105,106], and PGC-1α, while concurrently suppressing anabolic signaling via mTOR inhibition [107,108,109].

This molecular pattern triggers an adaptive shift toward enhanced autophagic flux—a cellular housekeeping mechanism essential for removing damaged organelles, mitigating oxidative stress, and preserving mitochondrial integrity [101]. The augmentation of mitophagy and restoration of mitochondrial biogenesis have been consistently observed in both in vivo and in vitro models, including in tissues that do not express SGLT2, such as the myocardium. This implies that SGLT2 inhibitors may act directly on nutrient and redox sensors, independent of glycosuria or systemic metabolic changes [101].

The net effect is the preservation of cardiomyocyte viability, attenuation of fibrosis, and improvement in myocardial structure and function [101]. These benefits align closely with the pathophysiologic mechanisms driving AS progression, which is marked by energy depletion, oxidative stress, and maladaptive remodeling [110,111].

Supporting this view, Yue et al. [84] demonstrated that empagliflozin downregulates fatty acid synthesis enzymes (FASN, SCD3, ACSL1, ACSL5) and improves metabolic balance in obese mice. In a rat model of supravalvular AS, Urbano Pagan et al. [95] showed that empagliflozin ameliorates left ventricular remodeling, enhances antioxidant defenses, and improves diastolic function—effects closely linked to metabolic remodeling and suppression of inflammatory mediators.

Taken together, these findings suggest that SGLT2 inhibitors not only optimize substrate utilization but also restore energetic and proteostatic homeostasis via autophagy-mediated stress resolution mechanisms. This multifaceted role provides a compelling mechanistic rationale for their evaluation in AS, where mitochondrial dysfunction, impaired energy signaling, and maladaptive hypertrophy are central to disease progression.

### 3.5. Cardiac Remodeling and Electrophysiologic Effects

A key contributor to morbidity in AS is myocardial remodeling and the associated risk of arrhythmias. Wen et al. [85] utilized a transverse aortic constriction (TAC) model to demonstrate that empagliflozin restored ejection fraction, reduced hypertrophy, and reversed calcium-handling abnormalities. Electrophysiologic remodeling was improved through a reduction in late sodium current (INaL), CaMKII phosphorylation, and Nav1.5 expression, while calcium transient alternans and action potential duration were normalized [85].

### 3.6. Effects on Erythropoiesis

Anemia is increasingly recognized as a clinically meaningful comorbidity in AS, portending worse outcomes across the disease continuum. In a landmark analysis of 7292 patients with severe AS, anemia was independently associated with a 75% higher risk of all-cause mortality and a 42% increase in sudden cardiac death (SCD), even after multivariable adjustment [112]. These findings are further corroborated by a recent meta-analysis involving over 12,500 patients undergoing transcatheter aortic valve replacement (TAVR), wherein anemia predicted heightened risks of periprocedural transfusion, acute kidney injury, and both short- and mid-term mortality [113]. Mechanistically, anemia exacerbates myocardial oxygen supply–demand mismatch and may amplify AS-related left ventricular hypertrophy, neurohormonal activation, and frailty [113]. Despite its prevalence and adverse prognostic weight, anemia remains under-targeted in AS management algorithms.

Intriguingly, SGLT2 inhibitors—beyond their glycemic and cardiorenal effects—have demonstrated consistent capacity to stimulate erythropoiesis. In a mechanistic substudy of the EMPA-HEART trial, empagliflozin induced early and sustained increases in erythropoietin, hematocrit, and red blood cell mass in patients with type 2 diabetes and coronary artery disease [114]. The EMPATROPISM-FE trial further showed that empagliflozin enhanced myocardial iron content and activated hematopoiesis in non-diabetic patients with heart failure, with reverse remodeling being particularly pronounced in those with baseline anemia [115]. Complementing these clinical observations, Packer has proposed a multifaceted model in which SGLT2 inhibitors suppress hepcidin, enhance iron mobilization, and upregulate hepatic and renal hypoxia-inducible factor-2α (HIF-2α), thereby augmenting erythropoietin synthesis via both classical and SIRT1-dependent nutrient-sensing pathways [116]. These mechanisms are substantiated by a meta-analysis of 40 randomized trials encompassing >21,000 patients, which confirmed that SGLT2 inhibition significantly increases hematocrit, with effects most pronounced for empagliflozin and canagliflozin [117]. Collectively, these findings unveil a previously underappreciated therapeutic axis wherein SGLT2 inhibitors may mitigate anemia-associated risk in AS through mechanisms distinct from volume contraction or hemoconcentration, with potential implications for improving myocardial oxygenation and delaying decompensation in this high-risk population.

## 4. Evidence of SGLT2 Expression in Aortic Valve Stenosis and Clinical Outcomes

Emerging evidence suggests that the pathophysiology of AS extends beyond valvular obstruction to encompass profound myocardial remodeling—processes in which SGLT2 expression may play a pivotal role.

The BIO-AS study [98] provided the first direct evidence that SGLT2 is expressed in the human myocardium in the context of AS, even in patients without diabetes (Table 2). This multicenter biomarker study included 45 patients with severe AS—classified as either high-gradient (HG) or low-flow low-gradient (LF–LG)—and 10 controls undergoing non-valvular cardiac surgery [98]. Compared with controls, myocardial SGLT2 gene and protein expression were significantly elevated in patients with AS, with the highest levels observed in those with the LF–LG phenotype [98]. These findings remained robust after adjusting for potential confounders, including age, sex, BMI, diabetes, AH, and CAD. Notably, SGLT2 expression positively correlated with key mediators of myocardial fibrosis (TGF-β: r = 0.72; collagen: r = 0.73), inflammation (IL-6: r = 0.68; NF-κB: r = 0.36), and was inversely correlated with antioxidant defense mechanisms (SOD2: r = −0.38) [98]. Furthermore, myocardial expression of GLUT4 and PPAR-γ was significantly upregulated, while PPAR-α was downregulated, indicating a shift toward an energetically inefficient, pro-fibrotic metabolic profile in AS, especially in LF–LG patients [98]. These molecular changes align with the maladaptive cardiac remodeling observed in advanced AS and position SGLT2 not only as a metabolic transporter but also as a central node linking inflammation, fibrosis, and energetic dysfunction in the diseased myocardium [98].

These mechanistic insights gain clinical relevance when considered alongside the findings from Shah et al. [118], who conducted a large retrospective target trial emulation using electronic health records from over 11,000 patients with nonsevere AS. In this analysis, SGLT2 inhibitor use was associated with a significantly lower risk of progression to severe AS (HR: 0.61; 95% CI: 0.39–0.94; *p* = 0.03), with an even greater risk reduction observed with longer treatment duration [118]. Annual hemodynamic deterioration—measured by the change in AVA and peak transvalvular velocity—was also slower in SGLT2 inhibitor users [118]. These findings suggest that SGLT2 inhibitors may influence disease trajectory even before the onset of symptomatic severe AS, potentially through modulation of the fibrotic and inflammatory pathways identified in the BIO-AS study [118].

Together, the translational and clinical data converge on a compelling hypothesis: SGLT2 overexpression may be both a marker and a driver of adverse myocardial remodeling in AS, and pharmacologic inhibition of this pathway may serve as a strategy to attenuate disease progression and improve outcomes across the AS continuum.

## 5. Therapeutic Potential in the Context of TAVI and AVR

While TAVI and surgical AVR have revolutionized the management of severe AS, these structural interventions primarily address valvular obstruction without reversing the chronic myocardial remodeling that often precedes intervention [123,124,125]. A substantial proportion of patients—particularly those with pre-existing myocardial fibrosis, low-flow phenotypes, or heart failure with preserved or mildly reduced ejection fraction—continue to experience adverse clinical outcomes even after successful valve replacement [126,127,128,129]. Post-procedural heart failure hospitalizations, impaired left ventricular recovery, and residual cardiac damage remain common, highlighting the need for adjunctive therapies that target the myocardial substrate directly [130,131,132,133].

Recent observational and randomized studies have begun to explore the role of SGLT2 inhibitors in this setting. The DapaTAVI trial [121,134] was a multicenter, randomized controlled study evaluating the efficacy and safety of dapagliflozin in older adults with severe aortic stenosis undergoing TAVI and at high risk for heart failure events [121]. A total of 1222 patients (mean age 82 years; 49% women) with prior heart failure and at least one additional high-risk feature—reduced ejection fraction (≤40%), diabetes mellitus, or moderate renal impairment—were randomized to receive either dapagliflozin (10 mg daily) or standard care [121]. At 1 year, dapagliflozin significantly reduced the incidence of the primary composite endpoint of all-cause mortality or worsening heart failure (15.0% vs. 20.1%; hazard ratio [HR]: 0.72; 95% CI: 0.55–0.95; *p* = 0.02) [121]. Notably, the risk of worsening heart failure—defined as hospitalization or urgent visits requiring intravenous diuretics—was significantly lower in the dapagliflozin group (9.4% vs. 14.4%; subhazard ratio: 0.63; 95% CI: 0.45–0.88). The benefit extended across key subgroups, including patients with preserved ejection fraction and those over 80 years of age [121]. Dapagliflozin was generally well tolerated, with a modestly increased incidence of genital infections and hypotension [121]. These findings provide the first randomized evidence supporting SGLT2 inhibition as an effective adjunctive strategy to reduce post-TAVI heart failure burden in high-risk patients.

These randomized results are further supported by real-world evidence [120]. In a multicenter international registry, Paolisso et al. [120] evaluated the effects of SGLT2 inhibitors in 311 patients with type 2 diabetes, severe AS, LVEF < 50%, and extra-valvular cardiac damage (EVCD) undergoing TAVI. Among the cohort, 24% were discharged on an SGLT2 inhibitor (empagliflozin or dapagliflozin). Despite a more adverse baseline myocardial profile, these patients exhibited significantly greater LV recovery—particularly among those with LVEF ≤ 30%—and a higher proportion showed stabilization or improvement in the EVCD stage compared to those not on SGLT2 inhibitors (92.7% vs. 78.7%, *p* = 0.018) [120]. Over a median 2-year follow-up, SGLT2 inhibitors use was independently associated with a reduced risk of major adverse cardiovascular events (HR: 0.45), all-cause mortality (HR: 0.51), and heart failure hospitalization (HR: 0.40) [120]. A landmark analysis confirmed that clinical benefits emerged beyond the initial 30-day procedural window, reinforcing the hypothesis that SGLT2 inhibitors may facilitate reverse remodeling and durable myocardial protection in patients with severe AS following TAVI [120].

Additional support for the therapeutic potential of SGLT2 inhibitors in AS comes from the EASTER-HF study [119], a retrospective observational analysis evaluating the role of empagliflozin in patients with severe degenerative AS (DAS) and HF prior to AVR. This study included 40 patients—20 treated with empagliflozin (10 mg daily) in addition to standard of care (SOC) and 20 receiving SOC alone—over a median preoperative period of approximately 2.7 months [119]. Despite the small sample size, empagliflozin use was associated with a significant 73% relative risk reduction in the composite outcome of cardiac death or HHF at 6 months (RR: 0.27; *p* = 0.022) [119]. Improvements in surrogate markers were also observed, including a 3.5% increase in LVEF and a marked reduction in NT-proBNP levels (−3975 pg/mL, *p* < 0.001) [119]. Notably, benefits were observed across a broad LVEF range, with most patients classified as having HFpEF [119]. The findings suggest that short-term preprocedural SGLT2 inhibitor therapy may serve as an effective bridging strategy to AVR, potentially attenuating myocardial stress and improving early postoperative outcomes [119]. Although limited by its retrospective, single-center design, this study provides a mechanistic rationale and clinical signal for integrating SGLT2 inhibitors into the peri-AVR management of high-risk AS patients.

Collectively, these findings underscore the emerging role of SGLT2 inhibitors as a promising adjunctive strategy in the management of AS, capable not only of improving post-procedural outcomes but also of intervening in the cardiometabolic remodeling process that underlies disease progression and long-term morbidity.

## 6. Future Directions

While accumulating evidence supports the cardioprotective effects of SGLT2 inhibitors across various heart failure phenotypes [135,136,137,138,139,140] and increasingly within aortic stenosis (AS), robust data from prospective, AS-specific randomized trials remain limited. To date, most trials investigating SGLT2 inhibitors in patients with HFpEF—such as EMPEROR-Preserved [21] and DELIVER [22]—have excluded patients with significant valvular disease, limiting generalizability to the AS population. The recent DapaTAVI trial [121] represents a major advancement, offering the first randomized evidence of clinical benefit in post-TAVI patients; however, that study focused on high-risk patients with established heart failure rather than early-stage AS or asymptomatic populations.

Beyond DapaTAVI, no other completed or ongoing randomized trials have yet evaluated the effect of SGLT2 inhibitors in patients undergoing TAVI. Nonetheless, observational datasets, including those from large registries and real-world cohorts, continue to suggest potential clinical benefits. Additionally, mechanistic insights from experimental and translational studies may guide the design of future trials, particularly those focused on myocardial fibrosis, metabolic remodeling, and inflammation in AS patients with overlapping HF phenotypes.

Although myosin inhibitors, particularly mavacamten, have demonstrated clinical benefit in patients with hypertrophic cardiomyopathy by targeting hypercontractility and improving diastolic function [141,142], their role in valvular aortic stenosis remains unexplored. This likely reflects fundamental differences in the underlying pathophysiology, namely, fixed valvular obstruction and pressure overload in AS versus sarcomeric hypercontractility in hypertrophic cardiomyopathy. Nonetheless, the success of myosin inhibition as a disease-modifying strategy underscores the therapeutic potential of sarcomere-targeted agents in reversing maladaptive remodeling [143,144]. Future translational studies may explore whether such mechanisms could be leveraged to modulate myocardial responses in AS, particularly in the pre-intervention phase or in patients with discordant valve gradients and preserved ejection fraction.

Importantly, as efforts to establish the role of SGLT2 inhibitors in AS progress, it will be critical to consider their integration with existing pharmacologic strategies. Table 3 summarizes the available clinical evidence for commonly used therapies in AS, including beta-blockers, renin–angiotensin system (RAS) inhibitors, calcium channel blockers, and nitrates. While some agents offer benefits in selected populations, the potential additive or synergistic effects of SGLT2 inhibitors remain unexplored. Future trials should address these interactions to optimize treatment strategies and define responsive subgroups.

Currently, no medical therapy alters the natural course of AS. As outlined in the 2021 ESC/EACTS Guidelines [165], statins have shown no clinical benefit despite encouraging preclinical data [12], and trials targeting calcium metabolism are ongoing. Pharmacologic management remains guided by comorbidities, particularly heart failure, rather than AS-specific targets. Standard therapies such as beta-blockers and RAS inhibitors are commonly employed in patients with coexisting heart failure, including those awaiting or unsuitable for valve replacement. ACE inhibitors are considered safe when blood pressure is monitored and may confer myocardial benefit before or after valve intervention [151]. Thus, while SGLT2 inhibitor studies often involve patients receiving conventional therapies, these represent background heart failure management rather than disease-modifying treatment for AS [166].

Looking forward, it is imperative that future investigations prioritize the development of prospective, AS-specific randomized controlled trials across the disease continuum—from patients with moderate AS to those in the post-AVR setting. These studies should aim to determine the optimal timing, duration, and patient selection strategies for SGLT2 inhibitor therapy. Biomarkers such as myocardial SGLT2 expression, circulating fibrosis markers (e.g., galectin-3, ST2), and advanced imaging metrics of reverse remodeling may serve as tools to enhance therapeutic precision. Collectively, these efforts will be essential to establishing whether SGLT2 inhibitors can be positioned not merely as adjuncts but as disease-modifying agents in the management of AS.

## 7. Conclusions

Based on preliminary evidence, SGLT2 inhibitors represent a promising adjunct in the management of AS by targeting key elements of myocardial pathobiology—fibrosis, inflammation, oxidative stress, and metabolic dysfunction. Emerging evidence also points to potential valvular benefits, suggesting a dual impact on both the ventricle and the valve. Integrating molecular and clinical insights will be essential to redefine therapeutic strategies and move toward truly disease-modifying interventions in AS.

## Figures and Tables

**Table 1:** Mechanistic evidence supporting the cardiovascular and valvular effects of SGLT2 inhibitors.

Author, Year	Model/Study Design	Key Molecular Targets or Pathways	Main Findings	Implications for AS
Zheng et al., 2025 [82]	Retrospective cohort; 4964 T2DM patients (1942 SGLT2i users)	Inflammation (CRP, neutrophils, lymphocytes), oxidative stress (uric acid, bilirubin, GGT)	SGLT2i use was associated with a 9% lower risk of aortic aneurysm (adjusted HR: 0.91, *p* = 0.001); effects mediated in part by reductions in inflammatory and oxidative stress markers. Dapagliflozin and empagliflozin showed the strongest risk reduction.	Supports anti-inflammatory and antioxidative effects of SGLT2i; suggests vascular benefits may extend to aortic pathology, relevant for AS patients with vascular remodeling and inflammation.
Alsereidi et al., 2024 [83]	In vitro (cardiomyocytes, aortic endothelial cells, SC-β cells)	AKT/PI3K, SGLT2, NHE1, GLUT1, NRF2, MAPK, NF-κB, NLRP3	Dapagliflozin attenuated ISO-induced cardiomyocyte hypertrophy and inflammation via AKT/PI3K activation, reduced ROS, and suppressed NLRP3 inflammasome activity. In AECs, it restored eNOS expression, suppressed TNFα-induced NF-κB and VCAM/ICAM expression, and reduced GRP78 (ER stress marker). In SC-β cells, DAPA enhanced insulin functionality and MAFA expression while reducing NHE1 and GRP78 expression under inflammatory stress.	Demonstrates multifaceted anti-inflammatory, antioxidant, and anti-hypertrophic effects of SGLT2i across relevant cardiovascular and endocrine cell types, supporting their therapeutic potential in AS-associated myocardial and vascular remodeling.
Yue et al., 2024 [84]	Obese mice model; aortic proteomics with empagliflozin intervention (10 mg/kg/day for 12 weeks)	Fatty acid metabolism (FASN, SCD3, ACSL1, ACSL5), mitochondrial energetics, aortic stiffness (PWV)	Empagliflozin reduced expression of FASN, SCD3, ACSL1, and ACSL5 in the aorta; improved lipid/glucose profile; attenuated aortic stiffness (PWV ↓), collagen deposition, and endothelial injury. Proteomics confirmed shift in metabolic pathways.	Suggests empagliflozin may modulate aortic fatty acid metabolism, reduce stiffness, and preserve vascular integrity—mechanisms relevant to AS progression and its vascular complications.
Wen et al., 2024 [85]	Murine model; transverse aortic constriction (TAC) with Langendorff-perfused heart analysis	INaL (late Na+ current), p-CaMKII, Nav1.5, NCX, RyR2, Ca^2+^ transient alternans, CaTD80, TTP100	Empagliflozin attenuated TAC-induced cardiac hypertrophy, restored ejection fraction and fractional shortening, and reversed electrophysiologic remodeling (shortened APD80, improved Ca^2+^ handling, reduced arrhythmogenicity). It reduced p-CaMKII and Nav1.5 expression and improved Ca^2+^ transient kinetics and alternans under isoproterenol stress.	Demonstrates that empagliflozin counteracts pressure overload-induced cardiac remodeling, restores Ca^2+^ homeostasis, and prevents ventricular arrhythmias—findings highly relevant to AS-associated hypertrophy and decompensation.
Chandrasekar et al., 2023 [86]	In vitro (human aortic SMCs exposed to OxLDL); molecular assays and confocal microscopy	RECK, MMP2/9, miR-30b, NF-κB, CT-1, LIFR, gp130	Empagliflozin reversed OxLDL-induced miR-30b expression and RECK suppression; inhibited MMP2/9 activation, SMC proliferation/migration, and inflammatory phenotype. Also inhibited CT-1-mediated mitogenic effects via LIFR/gp130.	Highlights empagliflozin’s direct vascular protective effects in modulating matrix remodeling, inflammation, and oxidative stress—relevant to aortic wall changes and valvular sclerosis in AS.
Campeau et al., 2024 [87]	In vitro (human aortic endothelial cells exposed to tunicamycin-induced ER stress)	TXNIP, NLRP3, CHOP, phospho-eIF2α/eIF2α, NRF2	Empagliflozin (50–100 µM) reduced tunicamycin-induced ER stress and inflammation in ECs by downregulating CHOP, TXNIP, and NLRP3 and dampening NRF2 nuclear translocation. Effects were dose-dependent.	Supports a role for SGLT2i in reducing endothelial ER stress and inflammasome activation, mechanisms implicated in valvular inflammation and aortic endothelial dysfunction in AS.
Kawade et al., 2023 [88]	Diet-induced obese mice; luseogliflozin treatment with free or paired feeding	SOD2, ROS, ICAM-1, VCAM-1, MCP-1, FFA-induced oxidative stress	Luseogliflozin improved endothelial function by increasing SOD2 expression and reducing ROS in the thoracic aorta. It reversed FFA-induced endothelial dysfunction and metabolic abnormalities under caloric restriction.	Provides mechanistic evidence that SGLT2i enhances vascular antioxidant defenses and restores endothelial function—critical processes in AS-related aortic remodeling.
Liu et al., 2022 [89]	Murine model of AAA (PPE-induced); dapagliflozin 1 or 5 mg/kg for 14 days	Inflammation (macrophages, T/B cells), MMP2/9, angiogenesis (CD31), SMCs	Dapagliflozin reduced aneurysm formation and progression by decreasing aortic leukocyte infiltration, attenuating MMP2/9 expression, preserving SMCs, and reducing mural angiogenesis. Also limited progression of existing AAAs.	Reinforces anti-inflammatory, anti-proteolytic, and vascular-stabilizing effects of SGLT2i; may translate to benefits in AS-related aortic wall inflammation and remodeling.
Ashry et al., 2021 [90]	Hypercholesterolemic rabbit model; canagliflozin (10 mg/kg/day) for 4 weeks	Oxidative stress (SOD, MDA, GSH), NOx, CRP, PPARγ, endothelial function	Canagliflozin improved lipid profile, decreased CRP and oxidative markers (MDA, NOx), restored antioxidant enzymes (SOD, GSH), and enhanced acetylcholine-induced aortic relaxation. It also reduced aortic intima/media ratio and atherosclerotic lesion area.	Provides evidence for vascular protective effects of SGLT2i via anti-inflammatory, antioxidative, and endothelial mechanisms, supporting their relevance in AS-associated vascular remodeling.
Sukhanov et al., 2021 [91]	In vitro (human aortic SMC); IL-17A stimulation ± empagliflozin (1 µM)	TRAF3IP2, ROS, NLRP3, caspase-1, IL-1β, IL-18	Empagliflozin inhibited IL-17A-induced oxidative stress, NLRP3 expression, caspase-1 activation, and IL-1β/IL-18 secretion in SMCs. Reduced SMC proliferation and migration were observed, independent of glucose levels.	Provides strong mechanistic support for anti-inflammatory and anti-remodeling effects of empagliflozin in aortic smooth muscle—a key cellular contributor to vascular thickening and stiffness in AS.
Ortega et al., 2019 [92]	ApoE^−/−^ mice infused with Ang II; empagliflozin 1 or 3 mg/kg/day for 28 days	CCL-2, CCL-5, VEGF, MMP-2/9, TIMP-1, p38 MAPK, NF-κB, VCAM-1, ICAM-1	Empagliflozin significantly reduced Ang II induced dissecting AAA by limiting aortic dilation, elastin degradation, macrophage infiltration, and neovascularization. It downregulated inflammatory chemokines, MMPs, and endothelial adhesion molecules. Also inhibited activation of p38 MAPK and NF-κB pathways and preserved SMCs.	Demonstrates robust vascular anti-inflammatory and anti-proteolytic effects of empagliflozin; supports potential role in modulating aortic wall integrity and inflammation relevant to AS progression and complications.
El-Daly et al., 2018 [93]	In vitro (mouse aortic rings, endothelial cells); hyperglycemia-induced dysfunction model	SGLT2, ROS, NADPH oxidase, Src, EGFR, PKC, Rho-kinase, eNOS, PAR2	Hyperglycemia impaired PAR2-mediated vasodilation via ROS generation and downstream signaling. Empagliflozin preserved endothelial NO-dependent vasodilation by inhibiting SGLT2-mediated glucose uptake, reducing ROS, and modulating NADPH oxidase, EGFR/Src/PKC/Rho-kinase signaling.	Highlights aortic endothelial expression of SGLT2 and provides mechanistic basis for vascular protection via antioxidative, eNOS-preserving pathways—highly relevant for AS-associated endothelial dysfunction.
Solini et al., 2017 [94]	Pilot clinical study; 16 T2DM patients treated with dapagliflozin vs. 10 on HCT (2-day intervention)	Endothelial function (FMD), aortic stiffness (PWV), renal resistive index (RI), oxidative stress (urinary isoprostanes)	Dapagliflozin acutely improved FMD (2.8% → 4.0%), reduced PWV (10.1 → 8.9 m/s), and decreased RI (0.62 → 0.59). These effects occurred independently of natriuresis or blood glucose changes and were associated with a reduction in urinary isoprostanes. No similar vascular improvements were seen with HCT.	Provides early clinical evidence that SGLT2i enhances vascular function and reduces oxidative stress—mechanisms that may contribute to improved aortic and valvular compliance in AS.
Urbano Pagan et al., 2023 [95]	Rat model of AS (supravalvular banding); empagliflozin 10 mg/kg/day for 8 weeks	Collagen I/III, IL-6, NF-κB (p65), glutathione peroxidase, MMP-2, catalase, lipid hydroperoxides	Empagliflozin improved LV remodeling and diastolic function and reduced interstitial fibrosis, oxidative stress, and inflammatory signaling (IL-6, p65 NF-κB). It enhanced antioxidant enzyme activity (GPx), modulated MMP-2 activation, and reduced myocardial collagen content.	Provides direct in vivo evidence that SGLT2i improves myocardial remodeling, oxidative balance, and diastolic function in AS—supporting disease-modifying potential in pressure overload-induced cardiac pathology.

Abbreviations: AECs, aortic endothelial cells; AS, aortic stenosis; AAA, abdominal aortic aneurysm; ACSL, acyl-CoA synthetase long-chain; CaMKII, Ca^2+^/calmodulin-dependent protein kinase II; CHOP, C/EBP homologous protein; CT-1, cardiotrophin-1; ECs, endothelial cells; EGFR, epidermal growth factor receptor; eNOS, endothelial nitric oxide synthase; FASN, fatty acid synthase; FFA, free fatty acid; FMD, flow-mediated dilation; GGT, gamma-glutamyl transferase; GSH, glutathione; ICAM-1, intercellular adhesion molecule-1; IL, interleukin; LIFR, leukemia inhibitory factor receptor; MAPK, mitogen-activated protein kinase; MAFA, v-maf musculoaponeurotic fibrosarcoma oncogene homolog A; MCP-1, monocyte chemoattractant protein-1; MDA, malondialdehyde; MMP, matrix metalloproteinase; NADPH, nicotinamide adenine dinucleotide phosphate; NF-κB, nuclear factor kappa-light-chain-enhancer of activated B cells; NOx, nitric oxide metabolites; NRF2, nuclear factor erythroid 2–related factor 2; PAR2, protease-activated receptor-2; PPAR, peroxisome proliferator-activated receptor; PWV, pulse wave velocity; RECK, reversion-inducing cysteine-rich protein with Kazal motifs; RI, renal resistive index; ROS, reactive oxygen species; SC-β, stem cell-derived β cells; SGLT2i, sodium–glucose cotransporter 2 inhibitor; SMCs, smooth muscle cells; SOD2, superoxide dismutase 2; T2DM, type 2 diabetes mellitus; TNFα, tumor necrosis factor alpha; TXNIP, thioredoxin-interacting protein; VCAM-1, vascular cell adhesion molecule-1; VEGF, vascular endothelial growth factor.

**Table 2:** Overview of studies evaluating SGLT2 Inhibitors in the context of aortic valve disease and TAVI.

Author, Year	Study Design/Population	SGLT2 Inhibitor	Primary Endpoint(s)	Main Findings
Shah et al., 2025 [118]	Retrospective, multicenter target trial emulation; 11,698 patients with nonsevere AS (458 on SGLT2i, 11,240 not on SGLT2i)	Various (not specified individually)	Progression to severe AS	SGLT2i use was associated with a 39% lower risk of progression to severe AS (HR: 0.61; 95% CI: 0.39–0.94; *p* = 0.03); effect was stronger with longer exposure duration
Scisciola et al., 2024 [98]	Observational biomarker study; 45 patients with severe AS (HG and LF–LG) vs. 10 controls undergoing non-valvular cardiac surgery	Focus on endogenous myocardial SGLT2 expression	Myocardial SGLT2 gene/protein expression and association with markers of cardiac remodeling	SGLT2 gene and protein expression were markedly elevated in LF–LG AS patients versus HG AS and controls, independently of diabetes. Expression correlated positively with fibrosis markers (TGF-β, collagen), inflammation (IL-6, NF-κB), metabolic dysregulation (GLUT4, PPAR-γ ↑, PPAR-α ↓), and oxidative stress (↓ SOD2). SGLT2 expression independently predicted reduced LVEF and was associated with maladaptive myocardial remodeling.
Jariwala et al., 2024 [119]	Retrospective observational study; 40 patients with severe degenerative AS and heart failure (LVEF 30–80%), treated with empagliflozin (n = 20) or standard of care (n = 20) before AVR	Empagliflozin (10 mg daily)	Composite of heart failure hospitalization or cardiac death at 6 months	Empagliflozin significantly reduced 6-month HF hospitalization or death by 73% (RR: 0.27; *p* = 0.022). LVEF improved by +3.5%, and NT-proBNP decreased by 3975 pg/mL in the treatment group. Benefits observed across HFpEF and HFrEF phenotypes. No unexpected safety signals reported.
Paolisso et al., 2024 [120]	Multicenter observational registry; 311 diabetic patients with severe AS, LVEF < 50%, and extra-valvular cardiac damage (EVCD) undergoing TAVI	Empagliflozin or Dapagliflozin	Composite of all-cause death and HF hospitalization (MACE) at 2 years	SGLT2i users had significantly higher rates of LV recovery and more often showed stable or improved EVCD. At 2 years, SGLT2i use was independently associated with reduced MACE (HR: 0.45), all-cause death (HR: 0.51), and HF hospitalization (HR: 0.40). The benefit was most evident in patients with baseline LVEF ≤ 30%, and emerged after the first 30 days post-TAVI.
Raposeiras-Roubín et al., 2025 [121]	Randomized controlled trial (DapaTAVI); 1222 high-risk patients with severe AS undergoing TAVI, history of HF, and ≥1 risk factor (diabetes, renal dysfunction, or LVEF ≤ 40%)	Dapagliflozin (10 mg daily)	Composite of all-cause death or worsening heart failure at 1 year	Dapagliflozin significantly reduced the primary composite outcome (15.0% vs. 20.1%, HR: 0.72, *p* = 0.02). Significant reductions were observed in HF worsening (9.4% vs. 14.4%, SHR: 0.63), HF hospitalizations, and urgent visits. Benefits consistent across subgroups; genital infections and hypotension occurred more frequently with dapagliflozin.
Thakkar et al., 2024 [122]	Retrospective cohort study; 67,604 patients with severe AS undergoing TAVI (2009–2024) using TriNetX network; 827 SGLT2i users vs. 827 non-users after propensity matching	Various (not specified)	Composite of all-cause mortality and HF hospitalization	SGLT2i use associated with lower risk of composite outcome (HR: 0.783; *p* = 0.004), HF hospitalization (HR: 0.799; *p* = 0.01), and acute kidney injury (HR: 0.563; *p* = 0.006); no significant difference in mortality, MI, stroke, or pacemaker implantation.

Abbreviations: AS, aortic stenosis; AVR, aortic valve replacement; EVCD, extra-valvular cardiac damage; HF, heart failure; HG, high-gradient; HR, hazard ratio; LF–LG, low-flow, low-gradient; LVEF, left ventricular ejection fraction; MACE, major adverse cardiovascular events; NT-proBNP, N-terminal pro–B-type natriuretic peptide; SHR, subhazard ratio; SGLT2i, sodium–glucose cotransporter 2 inhibitor; TAVI, transcatheter aortic valve implantation.

**Table 3:** Summary of key clinical studies evaluating pharmacologic therapies in aortic stenosis: beta-blockers, RAS inhibitors, calcium channel blockers, and nitrates.

Study	Design	Population	Intervention	Results
Hansson et al., 2017 [145]	Randomized, double-blind, placebo-controlled trial	40 patients with asymptomatic moderate-severe aortic stenosis	Metoprolol (extended-release, 100 ± 53 mg/day) for 22 weeks	↓ Heart rate by 8 bpm, ↑ ejection time by 26 ms, ↓ peak AV gradient by 7 mmHg, ↓ mean AV gradient by 4 mmHg, ↓ valvuloarterial impedance by 0.5 mmHg/mL·m^2^, ↓ myocardial oxygen consumption by 12%; stroke volume preserved; well tolerated
Rossi et al., 2015 [146]	Retrospective observational study	113 patients with symptomatic severe aortic stenosis (mean age 82 ± 8 years, 45% male)	Beta-blocker therapy (atenolol 16%, carvedilol 19%, metoprolol 5%, bisoprolol 60%)	↓ all-cause mortality by 62% (HR 0.38, 95% CI 0.14–0.96, *p* = 0.04); mortality: 21% in BB group vs. 51% in non-BB group; effect consistent regardless of BAV status
Hansson et al., 2024 [147]	Nationwide retrospective registry study (SWEDEHEART)	11,849 patients undergoing isolated surgical aortic valve replacement (median follow-up: 5.4 years)	Beta-blocker therapy (cardioselective only, dispensed at 6 months post-SAVR)	Crude MACE rate: 6.5 vs. 5.1 events/100 pt-yrs with vs. without BB; adjusted HR for MACE: 1.14 (95% CI 1.05–1.23); no significant difference in all-cause death [HR 1.06 (0.98–1.15)], stroke [HR 1.07 (0.91–1.25)], or MI [HR 0.94 (0.71–1.25)]; association attenuated after adjusting for emerging comorbidities [HR 1.04 (0.95–1.14)]
Bang et al., 2017 [148]	Post hoc analysis of a randomized controlled trial (SEAS study)	1873 asymptomatic patients with mild to moderate aortic stenosis and preserved LVEF	Beta-blocker therapy at baseline (metoprolol 48%, bisoprolol 19%, atenolol 16%, others 17%)	↓ all-cause mortality by 50% (HR 0.5, 95% CI 0.3–0.7, *p* < 0.001); ↓ cardiovascular death by 60% (HR 0.4, 95% CI 0.2–0.7, *p* < 0.001); ↓ sudden cardiac death by 80% (HR 0.2, 95% CI 0.1–0.6, *p* = 0.004); confirmed in competing risk analyses
Shumkova et al., 2024 [149]	Observational cohort study	61 patients with decompensated HFpEF and moderate aortic stenosis (mean age 82.7 ± 7.6 years)	Beta-blocker use at hospital discharge	↓ all-cause mortality and HF hospitalization with beta-blocker use; HR for composite endpoint: 0.27 (95% CI 0.13–0.57, *p* < 0.01); survivors more likely discharged on BBs (66% vs. 34%, *p* < 0.05); better diastolic function (higher septal e′) associated with improved outcomes
Hopfgarten et al., 2024 [150]	Nationwide retrospective cohort study (SWEDEHEART registry)	4668 patients with heart failure undergoing aortic valve replacement (2008–2016)	Beta-blocker therapy post-AVR (75% exposure in both reduced and preserved LVEF groups)	↓ all-cause mortality in reduced LVEF: HR 0.81 (95% CI 0.71–0.92); no mortality benefit in preserved LVEF: HR 0.87 (95% CI 0.69–1.10); no significant reduction in HF hospitalization in either group
Bull et al., 2015 (RIAS trial) [151]	Randomized controlled trial	100 asymptomatic patients with moderate/severe AS (mean age ~69 years, EF > 50%)	Ramipril 10 mg/day vs. placebo for 12 months	↓ LV mass (−3.9 g vs. +4.5 g; *p* = 0.006), ↑ systolic tissue velocity (0.0 vs. −0.5 cm/s; *p* = 0.04), trend toward slower AV area reduction (0.0 vs. −0.2 cm^2^; *p* = 0.067), well tolerated; no significant difference in adverse events
Goel et al., 2014 [152]	Retrospective cohort study	1752 patients post-SAVR	ACEI/ARB after SAVR	↑ 10-year survival (69% vs. 53%; *p* < 0.001)
Ochiai et al., 2018 [153]	Retrospective multicenter cohort	560 patients post-TAVR	ACEI/ARB post-TAVR	↓ LV mass regression and 2-yr mortality (7.5% vs. 12.5%; *p* = 0.031)
Rodriguez-Gabella et al., 2019 [154]	Retrospective multicenter cohort	2785 patients post-TAVR	RAS blockade post-TAVR	↓ CV death (5.6% vs. 9.5%; *p* < 0.001), ↓ stroke and HF rehospitalization
Inohara et al., 2018 [155]	Retrospective registry analysis	21,312 patients post-TAVR	RAS inhibitor at discharge post-TAVR	↓ 1-year mortality (12.5% vs. 14.9%; HR 0.82), ↓ HF readmission
Chen et al., 2020 [156]	Retrospective cohort (PARTNER 2)	3979 patients in PARTNER 2 trial	Baseline ACEI/ARB before TAVR	↓ 2-yr all-cause mortality (18.6% vs. 27.5%; *p* < 0.0001)
Chockalingam et al., 2004 (SCOPE-AS) [157]	Randomized controlled trial	56 patients with symptomatic severe AS (NYHA III–IV)	Enalapril (2.5 to 10 mg bid) vs. placebo for 4 weeks	Enalapril improved 6-min walk (+72 m vs. +27 m, *p* = 0.003), Borg index (−1.4 vs. −0.7, *p* = 0.03), NYHA class; well tolerated in patients with preserved LVEF; hypotension in 3 patients with low-normal BP and LV dysfunction
Bang et al., 2014 [158]	Retrospective analysis of RCT (SEAS study)	1873 asymptomatic patients with mild-to-moderate AS (mean follow-up: 4.3 years)	ACEI/ARB (n = 769) vs. no RASI (n = 1104)	No difference in SCD (HR 1.19, *p* = 0.694), CV mortality (HR 1.05, *p* = 0.854), or all-cause mortality (HR 0.81, *p* = 0.281); ↓ LVMI progression (*p* = 0.040), ↑ systolic BP reduction (*p* = 0.001); results confirmed in propensity-matched and time-varying Cox analyses
Dalsgaard et al., 2014 [159]	Randomized controlled trial	44 patients with severe AS (32 symptomatic, 12 asymptomatic; EF > 50%)	Trandolapril (up to 2 mg/day) vs. placebo for 3 days and up to 8 weeks	↓ SBP (−14 ± 11 mmHg vs. −5 ± 13; *p* = 0.02), ↑ SAC (0.08 ± 0.16 vs. −0.05 ± 0.86 mL/m^2^/mmHg; *p* = 0.03), ↓ LVESV at follow-up (−7.8 vs. −0.5 mL; *p* = 0.04), ↓ NT-proBNP (−19 vs. 0.8 pmol/L; *p* = 0.04); no significant changes in CO, PCWP, gradients, or adverse events
Yamamoto et al., 2024 (CURRENT AS Registry-2) [160]	Prospective multicenter observational registry with propensity score matching	2460 patients with severe AS and hypertension (71.7% on CCBs)	Antihypertensive therapy with vs. without calcium channel blockers	3-year all-cause death or HF hospitalization: 38.3% (CCB) vs. 38.7% (no CCB); HR 0.94 (95% CI 0.77–1.15; *p* = 0.56); ↓ sudden death in CCB group (4.2% vs. 5.2%; HR 0.48, *p* = 0.04); syncope: 1.1% vs. 1.0% (*p* = 0.74); comparable outcomes across age, AVR strategy, and AS severity
Saeed et al., 2020 [161]	Retrospective observational cohort (EXTAS study)	314 asymptomatic patients with moderate or severe AS (25% on CCBs)	Calcium channel blocker use vs. non-use	↓ exercise time (8.3 vs. 10.1 min; *p* = 0.001); ↓ peak HR (120 vs. 138 bpm; *p* < 0.001); ↑ blunted BP response (49% vs. 33%; *p* = 0.013); ↑ all-cause mortality: 20.3% vs. 5.6%; HR 7.09 (95% CI 2.15–23.38; *p* = 0.001)
Miyahara et al., 2025 [162]	Retrospective observational study with propensity score matching	993 patients undergoing TAVI for severe AS (CCB use at discharge: 59.4%)	Calcium channel blocker use at discharge vs. non-use	Composite endpoint (death or HF hospitalization): HR 0.879; *p* = 0.409 (no significant difference); Subgroup with CAD showed improved prognosis with CCB use (*p* for interaction = 0.002); Median follow-up: 719 days
Claveau et al., 2015 [163]	Retrospective cohort study	195 ED episodes of acute pulmonary edema: 65 each with severe AS, moderate AS, and no AS	Sublingual or intravenous nitroglycerin in patients with and without AS	Clinically relevant hypotension: 26.2% (severe AS) vs. 23.1% (no AS); adjusted OR 0.99 (95% CI 0.41–2.41); Sustained SBP < 90 mmHg ≥ 30 min: 29.2% (severe AS), OR 2.34 (95% CI 0.91–6.01); No increased use of vasopressors or fluid boluses; in-hospital mortality: 15.4% (severe AS)
Costa et al., 2024 [164]	Single-center prospective observational study	113 patients with severe AS undergoing cardiac CT prior to TAVI	Sublingual nitroglycerin (0.5 mg) vs. no nitroglycerin before cardiac CT	SLN group: ↓ SBP by −17.4 ± 19.3 mmHg vs. −1.9 ± 20.0 mmHg in control (*p* = 0.009); only 2 patients in SLN group and 1 in control had SBP < 100 mmHg; no symptomatic hypotension; SLN considered safe for coronary assessment

Abbreviations: AS, aortic stenosis; SAVR, surgical aortic valve replacement; TAVR, transcatheter aortic valve replacement; BB, beta-blocker; RAS, renin–angiotensin system; ACEI, angiotensin-converting enzyme inhibitor; ARB, angiotensin receptor blocker; CCB, calcium channel blocker; SLN, sublingual nitroglycerin; SBP, systolic blood pressure; HR, hazard ratio; CI, confidence interval; LV, left ventricle; LVMI, left ventricular mass index; LVEF, left ventricular ejection fraction; LVESV, left ventricular end-systolic volume; HF, heart failure; CAD, coronary artery disease; AVR, aortic valve replacement; CV, cardiovascular; MACE, major adverse cardiovascular events.

## Data Availability

All data generated in this research are included within this article.

## Supplementary Materials

The supporting information can be downloaded at https://www.mdpi.com/article/10.3390/ijms26104494/s1.

## References

[1] Hibino M., Pandey A.K., Hibino H., Verma R., Aune D., Yanagawa B., Takami Y., Bhatt D.L., Attizzani G.F., Pelletier M.P. (2023). Mortality Trends of Aortic Stenosis in High-Income Countries from 2000 to 2020. Heart.

[2] Mensah G.A., Fuster V., Murray C.J.L., Roth G.A., Mensah G.A., Abate Y.H., Abbasian M., Abd-Allah F., Abdollahi A., Abdollahi M. (2023). Global Burden of Cardiovascular Diseases and Risks, 1990–2022. J. Am. Coll. Cardiol..

[3] Lindman B.R., Clavel M.-A., Mathieu P., Iung B., Lancellotti P., Otto C.M., Pibarot P. (2016). Calcific Aortic Stenosis. Nat. Rev. Dis. Primers.

[4] (2022). Corrigendum to: 2021 ESC/EACTS Guidelines for the Management of Valvular Heart Disease: Developed by the Task Force for the Management of Valvular Heart Disease of the European Society of Cardiology (ESC) and the European Association for Cardio-Thoracic. Eur. Heart J..

[5] Otto C.M., Nishimura R.A., Bonow R.O., Carabello B.A., Erwin J.P., Gentile F., Jneid H., Krieger E.V., Mack M., McLeod C. (2021). 2020 ACC/AHA Guideline for the Management of Patients with Valvular Heart Disease: Executive Summary: A Report of the American College of Cardiology/American Heart Association Joint Committee on Clinical Practice Guidelines. J. Am. Coll. Cardiol..

[6] Strom J.B., Playford D., Stewart S., Li S., Shen C., Xu J., Strange G. (2022). Increasing Risk of Mortality across the Spectrum of Aortic Stenosis Is Independent of Comorbidity & Treatment: An International, Parallel Cohort Study of 248,464 Patients. PLoS ONE.

[7] Yarbrough W.M., Mukherjee R., Ikonomidis J.S., Zile M.R., Spinale F.G. (2012). Myocardial Remodeling with Aortic Stenosis and after Aortic Valve Replacement: Mechanisms and Future Prognostic Implications. J. Thorac. Cardiovasc. Surg..

[8] Rader F., Sachdev E., Arsanjani R., Siegel R.J. (2015). Left Ventricular Hypertrophy in Valvular Aortic Stenosis: Mechanisms and Clinical Implications. Am. J. Med..

[9] White M., Baral R., Ryding A., Tsampasian V., Ravindrarajah T., Garg P., Koskinas K.C., Clark A., Vassiliou V.S. (2021). Biomarkers Associated with Mortality in Aortic Stenosis: A Systematic Review and Meta-Analysis. Med. Sci..

[10] Beerkens F.J., Tang G.H.L., Kini A.S., Lerakis S., Dangas G.D., Mehran R., Khera S., Goldman M., Fuster V., Bhatt D.L. (2025). Transcatheter Aortic Valve Replacement Beyond Severe Aortic Stenosis: JACC State-of-the-Art Review. J. Am. Coll. Cardiol..

[11] Lindman B.R., Lindenfeld J. (2021). Prevention and Mitigation of Heart Failure in the Treatment of Calcific Aortic Stenosis: A Unifying Therapeutic Principle. JAMA Cardiol..

[12] Rossebø A.B., Pedersen T.R., Boman K., Brudi P., Chambers J.B., Egstrup K., Gerdts E., Gohlke-Bärwolf C., Holme I., Kesäniemi Y.A. (2008). Intensive Lipid Lowering with Simvastatin and Ezetimibe in Aortic Stenosis. N. Engl. J. Med..

[13] Lindman B.R., Sukul D., Dweck M.R., Madhavan M.V., Arsenault B.J., Coylewright M., Merryman W.D., Newby D.E., Lewis J., Harrell F.E.J. (2021). Evaluating Medical Therapy for Calcific Aortic Stenosis: JACC State-of-the-Art Review. J. Am. Coll. Cardiol..

[14] Lindman B.R., Merryman W.D. (2021). Unloading the Stenotic Path to Identifying Medical Therapy for Calcific Aortic Valve Disease: Barriers and Opportunities. Circulation.

[15] Dayan V., Vignolo G., Magne J., Clavel M.-A., Mohty D., Pibarot P. (2015). Outcome and Impact of Aortic Valve Replacement in Patients with Preserved LVEF and Low-Gradient Aortic Stenosis. J. Am. Coll. Cardiol..

[16] Belmonte M., Paolisso P., Bertolone D.T., Viscusi M.M., Gallinoro E., de Oliveira E.K., Shumkova M., Beles M., Esposito G., Addeo L. (2024). Combined Cardiac Damage Staging by Echocardiography and Cardiac Catheterization in Patients with Clinically Significant Aortic Stenosis. Can. J. Cardiol..

[17] Ribeiro H.B., Lerakis S., Gilard M., Cavalcante J.L., Makkar R., Herrmann H.C., Windecker S., Enriquez-Sarano M., Cheema A.N., Nombela-Franco L. (2018). Transcatheter Aortic Valve Replacement in Patients with Low-Flow, Low-Gradient Aortic Stenosis: The TOPAS-TAVI Registry. J. Am. Coll. Cardiol..

[18] Nombela-Franco L., del Trigo M., Morrison-Polo G., Veiga G., Jimenez-Quevedo P., Abdul-Jawad Altisent O., Campelo-Parada F., Biagioni C., Puri R., DeLarochellière R. (2015). Incidence, Causes, and Predictors of Early (≤30 Days) and Late Unplanned Hospital Readmissions After Transcatheter Aortic Valve Replacement. JACC Cardiovasc. Interv..

[19] Karakasis P., Patoulias D., Giannakoulas G., Sagris M., Theofilis P., Fragakis N., Biondi-Zoccai G. (2024). Effect of Glucagon-like Peptide-1 Receptor Agonism on Aortic Valve Stenosis Risk: A Mendelian Randomization Analysis. J. Clin. Med..

[20] McMurray J.J.V., Solomon S.D., Inzucchi S.E., Køber L., Kosiborod M.N., Martinez F.A., Ponikowski P., Sabatine M.S., Anand I.S., Bělohlávek J. (2019). Dapagliflozin in Patients with Heart Failure and Reduced Ejection Fraction. N. Engl. J. Med..

[21] Anker S.D., Butler J., Filippatos G., Ferreira J.P., Bocchi E., Böhm M., Brunner–La Rocca H.-P., Choi D.-J., Chopra V., Chuquiure-Valenzuela E. (2021). Empagliflozin in Heart Failure with a Preserved Ejection Fraction. N. Engl. J. Med..

[22] Solomon S.D., McMurray J.J.V., Claggett B., Boer R.A.d., DeMets D., Hernandez A.F., Inzucchi S.E., Kosiborod M.N., Lam C.S.P., Martinez F. (2022). Dapagliflozin in Heart Failure with Mildly Reduced or Preserved Ejection Fraction. N. Engl. J. Med..

[23] Udell J.A., Jones W.S., Petrie M.C., Harrington J., Anker S.D., Bhatt D.L., Hernandez A.F., Butler J. (2022). Sodium Glucose Cotransporter-2 Inhibition for Acute Myocardial Infarction: JACC Review Topic of the Week. J. Am. Coll. Cardiol..

[24] von Lewinski D., Kolesnik E., Tripolt N.J., Pferschy P.N., Benedikt M., Wallner M., Alber H., Berger R., Lichtenauer M., Saely C.H. (2022). Empagliflozin in Acute Myocardial Infarction: The EMMY Trial. Eur. Heart J..

[25] Paolisso P., Bergamaschi L., Gragnano F., Gallinoro E., Cesaro A., Sardu C., Mileva N., Foà A., Armillotta M., Sansonetti A. (2023). Outcomes in Diabetic Patients Treated with SGLT2-Inhibitors with Acute Myocardial Infarction Undergoing PCI: The SGLT2-I AMI PROTECT Registry. Pharmacol. Res..

[26] Harrington J., Udell J.A., Jones W.S., Anker S.D., Bhatt D.L., Petrie M.C., Vedin O., Sumin M., Zwiener I., Hernandez A.F. (2022). Empagliflozin in Patients Post Myocardial Infarction Rationale and Design of the EMPACT-MI Trial. Am. Heart J..

[27] Stachteas P., Nasoufidou A., Karagiannidis E., Patoulias D., Karakasis P., Alexiou S., Samaras A., Zormpas G., Stavropoulos G., Tsalikakis D. (2024). The Role of Sodium Glucose Co-Transporter 2 Inhibitors in Atrial Fibrillation: A Comprehensive Review. J. Clin. Med..

[28] Lu J., Xie S., Deng Y., Xie X., Liu Y. (2022). Blocking the NLRP3 Inflammasome Reduces Osteogenic Calcification and M1 Macrophage Polarization in a Mouse Model of Calcified Aortic Valve Stenosis. Atherosclerosis.

[29] Li X.-X., Chen Z.-D., Sun X.-J., Yang Y.-Q., Jin H., Liu N.-F. (2024). Empagliflozin Ameliorates Vascular Calcification in Diabetic Mice through Inhibiting Bhlhe40-Dependent NLRP3 Inflammasome Activation. Acta Pharmacol. Sin..

[30] Karakasis P., Theofilis P., Vlachakis P.K., Korantzopoulos P., Patoulias D., Antoniadis A.P., Fragakis N. (2024). Atrial Fibrosis in Atrial Fibrillation: Mechanistic Insights, Diagnostic Challenges, and Emerging Therapeutic Targets. Int. J. Mol. Sci..

[31] Shah S.M., Shah J., Lakey S.M., Garg P., Ripley D.P. (2023). Pathophysiology, Emerging Techniques for the Assessment and Novel Treatment of Aortic Stenosis. Open Hear..

[32] Goody P.R., Hosen M.R., Christmann D., Niepmann S.T., Zietzer A., Adam M., Bönner F., Zimmer S., Nickenig G., Jansen F. (2020). Aortic Valve Stenosis: From Basic Mechanisms to Novel Therapeutic Targets. Arterioscler. Thromb. Vasc. Biol..

[33] Bing R., Cavalcante J.L., Everett R.J., Clavel M.-A., Newby D.E., Dweck M.R. (2019). Imaging and Impact of Myocardial Fibrosis in Aortic Stenosis. JACC Cardiovasc. Imaging.

[34] Delicce A.V., Makaryus A.N. (2025). Physiology, Frank Starling Law.

[35] Chambers J. (2006). The Left Ventricle in Aortic Stenosis: Evidence for the Use of ACE Inhibitors. Heart.

[36] Sun L., Chandra S., Sucosky P. (2012). Ex Vivo Evidence for the Contribution of Hemodynamic Shear Stress Abnormalities to the Early Pathogenesis of Calcific Bicuspid Aortic Valve Disease. PLoS ONE.

[37] Schwarz N.G., Kemp W.L. (2020). Educational Case: Aortic Valve Stenosis. Acad. Pathol..

[38] Zheng K.H., Tzolos E., Dweck M.R. (2020). Pathophysiology of Aortic Stenosis and Future Perspectives for Medical Therapy. Cardiol. Clin..

[39] Ajmone Marsan N., Delgado V., Shah D.J., Pellikka P., Bax J.J., Treibel T., Cavalcante J.L. (2023). Valvular Heart Disease: Shifting the Focus to the Myocardium. Eur. Heart J..

[40] Stassen J., Ewe S.H., Pio S.M., Pibarot P., Redfors B., Leipsic J., Genereux P., Van Mieghem N.M., Kuneman J.H., Makkar R. (2023). Managing Patients with Moderate Aortic Stenosis. JACC Cardiovasc. Imaging.

[41] Smucker M.L., Tedesco C.L., Manning S.B., Owen R.M., Feldman M.D. (1988). Demonstration of an Imbalance between Coronary Perfusion and Excessive Load as a Mechanism of Ischemia during Stress in Patients with Aortic Stenosis. Circulation.

[42] Treibel T.A., Badiani S., Lloyd G., Moon J.C. (2019). Multimodality Imaging Markers of Adverse Myocardial Remodeling in Aortic Stenosis. JACC Cardiovasc. Imaging.

[43] Chin C.W.L., Everett R.J., Kwiecinski J., Vesey A.T., Yeung E., Esson G., Jenkins W., Koo M., Mirsadraee S., White A.C. (2017). Myocardial Fibrosis and Cardiac Decompensation in Aortic Stenosis. JACC Cardiovasc. Imaging.

[44] Mahmod M., Francis J.M., Pal N., Lewis A., Dass S., De Silva R., Petrou M., Sayeed R., Westaby S., Robson M.D. (2014). Myocardial Perfusion and Oxygenation Are Impaired during Stress in Severe Aortic Stenosis and Correlate with Impaired Energetics and Subclinical Left Ventricular Dysfunction. J. Cardiovasc. Magn. Reson..

[45] Hjortnaes J., New S.E.P., Aikawa E. (2013). Visualizing Novel Concepts of Cardiovascular Calcification. Trends Cardiovasc. Med..

[46] New S.E.P., Goettsch C., Aikawa M., Marchini J.F., Shibasaki M., Yabusaki K., Libby P., Shanahan C.M., Croce K., Aikawa E. (2013). Macrophage-Derived Matrix Vesicles: An Alternative Novel Mechanism for Microcalcification in Atherosclerotic Plaques. Circ. Res..

[47] Rutkovskiy A., Malashicheva A., Sullivan G., Bogdanova M., Kostareva A., Stensløkken K.-O., Fiane A., Vaage J. (2017). Valve Interstitial Cells: The Key to Understanding the Pathophysiology of Heart Valve Calcification. J. Am. Heart Assoc..

[48] Michelena H.I., Prakash S.K., Della Corte A., Bissell M.M., Anavekar N., Mathieu P., Bossé Y., Limongelli G., Bossone E., Benson D.W. (2014). Bicuspid Aortic Valve: Identifying Knowledge Gaps and Rising to the Challenge from the International Bicuspid Aortic Valve Consortium (BAVCon). Circulation.

[49] Kerstjens-Frederikse W.S., van de Laar I.M.B.H., Vos Y.J., Verhagen J.M.A., Berger R.M.F., Lichtenbelt K.D., Klein Wassink-Ruiter J.S., van der Zwaag P.A., du Marchie Sarvaas G.J., Bergman K.A. (2016). Cardiovascular Malformations Caused by NOTCH1 Mutations Do Not Keep Left: Data on 428 Probands with Left-Sided CHD and Their Families. Genet. Med..

[50] Liu H., Chen Y.-G. (2022). The Interplay Between TGF-β Signaling and Cell Metabolism. Front. Cell Dev. Biol..

[51] Varshney R., Murphy B., Woolington S., Ghafoory S., Chen S., Robison T., Ahamed J. (2019). Inactivation of Platelet-Derived TGF-Β1 Attenuates Aortic Stenosis Progression in a Robust Murine Model. Blood Adv..

[52] Androshchuk V., Chehab O., Wilcox J., McDonaugh B., Montarello N., Rajani R., Prendergast B., Patterson T., Redwood S. (2024). Evolving Perspectives on Aortic Stenosis: The Increasing Importance of Evaluating the Right Ventricle before Aortic Valve Intervention. Front. Cardiovasc. Med..

[53] Scarsini R., Gallinoro E., Ancona M.B., Portolan L., Paolisso P., Springhetti P., Della Mora F., Mainardi A., Belmonte M., Moroni F. (2024). Characterisation of Coronary Microvascular Dysfunction in Patients with Severe Aortic Stenosis Undergoing TAVI. EuroIntervention.

[54] Lancellotti P., Nchimi A. (2017). Coronary Microvascular Reserve and Outcome in Aortic Stenosis: Pathophysiological Significance vs. Clinical Relevance. Eur. Heart J..

[55] Monga S., Valkovič L., Tyler D., Lygate C.A., Rider O., Myerson S.G., Neubauer S., Mahmod M. (2022). Insights Into the Metabolic Aspects of Aortic Stenosis with the Use of Magnetic Resonance Imaging. JACC Cardiovasc. Imaging.

[56] Cherpaz M., Meugnier E., Seillier G., Pozzi M., Pierrard R., Leboube S., Farhat F., Vola M., Obadia J.-F., Amaz C. (2024). Myocardial Transcriptomic Analysis of Diabetic Patients with Aortic Stenosis: Key Role for Mitochondrial Calcium Signaling. Cardiovasc. Diabetol..

[57] Wang K., Xie X., Hu X., Wang Z., Xia J., Wu Q. (2024). Stearic Acid Alleviates Aortic Medial Degeneration through Maintaining Mitochondrial Dynamics Homeostasis via Inhibiting JNK/MAPK Signaling. iScience.

[58] Sucosky P., Balachandran K., Elhammali A., Jo H., Yoganathan A.P. (2009). Altered Shear Stress Stimulates Upregulation of Endothelial VCAM-1 and ICAM-1 in a BMP-4– and TGF-β1–Dependent Pathway. Arterioscler. Thromb. Vasc. Biol..

[59] Timmerman L.A., Grego-Bessa J., Raya A., Bertrán E., Pérez-Pomares J.M., Díez J., Aranda S., Palomo S., McCormick F., Izpisúa-Belmonte J.C. (2004). Notch Promotes Epithelial-Mesenchymal Transition during Cardiac Development and Oncogenic Transformation. Genes Dev..

[60] Peeters F.E.C.M., Meex S.J.R., Dweck M.R., Aikawa E., Crijns H.J.G.M., Schurgers L.J., Kietselaer B.L.J.H. (2018). Calcific Aortic Valve Stenosis: Hard Disease in the Heart: A Biomolecular Approach towards Diagnosis and Treatment. Eur. Heart J..

[61] Lehti S., Käkelä R., Hörkkö S., Kummu O., Helske-Suihko S., Kupari M., Werkkala K., Kovanen P.T., Oörni K. (2013). Modified Lipoprotein-Derived Lipid Particles Accumulate in Human Stenotic Aortic Valves. PLoS ONE.

[62] Miller J.D., Chu Y., Brooks R.M., Richenbacher W.E., Peña-Silva R., Heistad D.D. (2008). Dysregulation of Antioxidant Mechanisms Contributes to Increased Oxidative Stress in Calcific Aortic Valvular Stenosis in Humans. J. Am. Coll. Cardiol..

[63] Galeone A., Brunetti G., Oranger A., Greco G., Di Benedetto A., Mori G., Colucci S., Zallone A., Paparella D., Grano M. (2013). Aortic Valvular Interstitial Cells Apoptosis and Calcification Are Mediated by TNF-Related Apoptosis-Inducing Ligand. Int. J. Cardiol..

[64] Isoda K., Matsuki T., Kondo H., Iwakura Y., Ohsuzu F. (2010). Deficiency of Interleukin-1 Receptor Antagonist Induces Aortic Valve Disease in BALB/c Mice. Arterioscler. Thromb. Vasc. Biol..

[65] Zhan Q., Song R., Zeng Q., Yao Q., Ao L., Xu D., Fullerton D.A., Meng X. (2015). Activation of TLR3 Induces Osteogenic Responses in Human Aortic Valve Interstitial Cells through the NF-ΚB and ERK1/2 Pathways. Int. J. Biol. Sci..

[66] West X.Z., Malinin N.L., Merkulova A.A., Tischenko M., Kerr B.A., Borden E.C., Podrez E.A., Salomon R.G., Byzova T.V. (2010). Oxidative Stress Induces Angiogenesis by Activating TLR2 with Novel Endogenous Ligands. Nature.

[67] Yutzey K.E., Demer L.L., Body S.C., Huggins G.S., Towler D.A., Giachelli C.M., Hofmann-Bowman M.A., Mortlock D.P., Rogers M.B., Sadeghi M.M. (2014). Calcific Aortic Valve Disease: A Consensus Summary from the Alliance of Investigators on Calcific Aortic Valve Disease. Arterioscler. Thromb. Vasc. Biol..

[68] Proudfoot D., Skepper J.N., Hegyi L., Bennett M.R., Shanahan C.M., Weissberg P.L. (2000). Apoptosis Regulates Human Vascular Calcification in Vitro: Evidence for Initiation of Vascular Calcification by Apoptotic Bodies. Circ. Res..

[69] Zebhi B., Lazkani M., Bark D.J. (2021). Calcific Aortic Stenosis—A Review on Acquired Mechanisms of the Disease and Treatments. Front. Cardiovasc. Med..

[70] Guo L., Du Y., Li H., He T., Yao L., Yang G., Yang X. (2025). Metabolites-Mediated Posttranslational Modifications in Cardiac Metabolic Remodeling: Implications for Disease Pathology and Therapeutic Potential. Metabolism.

[71] Vendrov A.E., Lozhkin A., Hayami T., Levin J., Chamon J.S.F., Abdel-Latif A., Runge M.S., Madamanchi N.R. (2024). Mitochondrial Dysfunction and Metabolic Reprogramming Induce Macrophage Pro-Inflammatory Phenotype Switch and Atherosclerosis Progression in Aging. Front. Immunol..

[72] Pal N., Acharjee A., Ament Z., Dent T., Yavari A., Mahmod M., Ariga R., West J., Steeples V., Cassar M. (2024). Metabolic Profiling of Aortic Stenosis and Hypertrophic Cardiomyopathy Identifies Mechanistic Contrasts in Substrate Utilization. FASEB J..

[73] Monga S., Valkovic L., Mahmod M., Myerson S.G., Neubauer S., Rider O.J. (2022). Characterisation of Metabolic Phenotype in Aortic Stenosis: Insights from a Multi-Parametric Cardiac Magnetic Resonance Study. Eur. Heart J..

[74] Peng G., Yan J., Chen L., Li L. (2023). Glycometabolism Reprogramming: Implications for Cardiovascular Diseases. Prog. Biophys. Mol. Biol..

[75] Pun-García A., Clemente-Moragón A., Villena-Gutierrez R., Gómez M., Sanz-Rosa D., Díaz-Guerra A., Prados B., Medina J.P., Montó F., Ivorra M.D. (2022). Beta-3 Adrenergic Receptor Overexpression Reverses Aortic Stenosis–Induced Heart Failure and Restores Balanced Mitochondrial Dynamics. Basic Res. Cardiol..

[76] Watson W.D., Green P.G., Lewis A.J.M., Arvidsson P., De Maria G.L., Arheden H., Heiberg E., Clarke W.T., Rodgers C.T., Valkovič L. (2023). Retained Metabolic Flexibility of the Failing Human Heart. Circulation.

[77] Monga S., Valkovič L., Myerson S.G., Neubauer S., Mahmod M., Rider O.J. (2023). Role of Cardiac Energetics in Aortic Stenosis Disease Progression: Identifying the High-Risk Metabolic Phenotype. Circ. Cardiovasc. Imaging.

[78] Elrakaybi A., Laubner K., Zhou Q., Hug M.J., Seufert J. (2022). Cardiovascular Protection by SGLT2 Inhibitors—Do Anti-Inflammatory Mechanisms Play a Role?. Mol. Metab..

[79] Mylonas N., Nikolaou P.E., Karakasis P., Stachteas P., Fragakis N., Andreadou I. (2024). Endothelial Protection by Sodium-Glucose Cotransporter 2 Inhibitors: A Literature Review of In Vitro and In Vivo Studies. Int. J. Mol. Sci..

[80] Karakasis P., Fragakis N., Kouskouras K., Karamitsos T., Patoulias D., Rizzo M. (2024). Sodium-Glucose Cotransporter-2 Inhibitors in Patients with Acute Coronary Syndrome: A Modern Cinderella?. Clin. Ther..

[81] Stachteas P., Karakasis P., Patoulias D., Clemenza F., Fragakis N., Rizzo M. (2023). The Effect of Sodium-Glucose Co-Transporter-2 Inhibitors on Markers of Subclinical Atherosclerosis. Ann. Med..

[82] Zheng R., Song W., Lu J., Yuan M., Sun X., Lu C. (2025). The Protective Role of SGLT2 Inhibitors on Aortic Aneurysm Mediated by Oxidative Stress and Inflammation in Type 2 Diabetes Mellitus. Cardiovasc. Diabetol..

[83] Alsereidi F.R., Khashim Z., Marzook H., Al-Rawi A.M., Salomon T., Almansoori M.K., Madkour M.M., Hamam A.M., Ramadan M.M., Peterson Q.P. (2024). Dapagliflozin Mitigates Cellular Stress and Inflammation through PI3K/AKT Pathway Modulation in Cardiomyocytes, Aortic Endothelial Cells, and Stem Cell-Derived β Cells. Cardiovasc. Diabetol..

[84] Yue L., Wang Y., Wang C., Niu S., Dong X., Guan Y., Chen S. (2024). Empagliflozin Improves Aortic Injury in Obese Mice by Regulating Fatty Acid Metabolism. Open Med..

[85] Wen Q., Zhang R., Ye K., Yang J., Shi H., Liu Z., Li Y., Liu T., Zhang S., Chen W. (2024). Empagliflozin Rescues Pro-Arrhythmic and Ca^2+^ Homeostatic Effects of Transverse Aortic Constriction in Intact Murine Hearts. Sci. Rep..

[86] Chandrasekar B., Mummidi S., DeMarco V.G., Higashi Y. (2023). Empagliflozin Reverses Oxidized LDL-Induced RECK Suppression, Cardiotrophin-1 Expression, MMP Activation, and Human Aortic Smooth Muscle Cell Proliferation and Migration. Mediat. Inflamm..

[87] Campeau M.-A., Leask R.L. (2024). Empagliflozin Reduces Endoplasmic Reticulum Stress Associated TXNIP/NLRP3 Activation in Tunicamycin-Stimulated Aortic Endothelial Cells. Naunyn-Schmiedeberg’s Arch. Pharmacol..

[88] Kawade S., Ogiso K., Shayo S.C., Obo T., Arimura A., Hashiguchi H., Deguchi T., Nishio Y. (2023). Luseogliflozin and Caloric Intake Restriction Increase Superoxide Dismutase 2 Expression, Promote Antioxidative Effects, and Attenuate Aortic Endothelial Dysfunction in Diet-Induced Obese Mice. J. Diabetes Investig..

[89] Liu H., Wei P., Fu W., Xia C., Li Y., Tian K., Li Y., Cheng D., Sun J., Xu Y. (2022). Dapagliflozin Ameliorates the Formation and Progression of Experimental Abdominal Aortic Aneurysms by Reducing Aortic Inflammation in Mice. Oxid. Med. Cell. Longev..

[90] Ashry N.A., Abdelaziz R.R., Suddek G.M., Saleh M.A. (2021). Canagliflozin Ameliorates Aortic and Hepatic Dysfunction in Dietary-Induced Hypercholesterolemia in the Rabbit. Life Sci..

[91] Sukhanov S., Higashi Y., Yoshida T., Mummidi S., Aroor A.R., Jeffrey Russell J., Bender S.B., DeMarco V.G., Chandrasekar B. (2021). The SGLT2 Inhibitor Empagliflozin Attenuates Interleukin-17A-Induced Human Aortic Smooth Muscle Cell Proliferation and Migration by Targeting TRAF3IP2/ROS/NLRP3/Caspase-1-Dependent IL-1β and IL-18 Secretion. Cell. Signal..

[92] Ortega R., Collado A., Selles F., Gonzalez-Navarro H., Sanz M.-J., Real J.T., Piqueras L. (2019). SGLT-2 (Sodium-Glucose Cotransporter 2) Inhibition Reduces Ang II (Angiotensin II)–Induced Dissecting Abdominal Aortic Aneurysm in ApoE (Apolipoprotein E) Knockout Mice. Arterioscler. Thromb. Vasc. Biol..

[93] El-Daly M., Pulakazhi Venu V.K., Saifeddine M., Mihara K., Kang S., Fedak P.W.M., Alston L.A., Hirota S.A., Ding H., Triggle C.R. (2018). Hyperglycaemic Impairment of PAR2-Mediated Vasodilation: Prevention by Inhibition of Aortic Endothelial Sodium-Glucose-Co-Transporter-2 and Minimizing Oxidative Stress. Vasc. Pharmacol..

[94] Solini A., Giannini L., Seghieri M., Vitolo E., Taddei S., Ghiadoni L., Bruno R.M. (2017). Dapagliflozin Acutely Improves Endothelial Dysfunction, Reduces Aortic Stiffness and Renal Resistive Index in Type 2 Diabetic Patients: A Pilot Study. Cardiovasc. Diabetol..

[95] Urbano Pagan L., Gomes M.J., Damatto F.C., Oliveira J.P.G., Gatto M., Souza L.M., Santos A.C.C., Borim P.A., Rodrigues E.A., Mota G.A.F. (2023). Effects of SGLT2 Inhibition on Cardiac Remodeling and Heart Failure Development in Rats with Aortic Stenosis. Eur. Heart J..

[96] Marfella R., Scisciola L., D’Onofrio N., Maiello C., Trotta M.C., Sardu C., Panarese I., Ferraraccio F., Capuano A., Barbieri M. (2022). Sodium-Glucose Cotransporter-2 (SGLT2) Expression in Diabetic and Non-Diabetic Failing Human Cardiomyocytes. Pharmacol. Res..

[97] Scisciola L., Taktaz F., Fontanella R.A., Pesapane A., Surina, Cataldo V., Ghosh P., Franzese M., Puocci A., Paolisso P. (2023). Targeting High Glucose-Induced Epigenetic Modifications at Cardiac Level: The Role of SGLT2 and SGLT2 Inhibitors. Cardiovasc. Diabetol..

[98] Scisciola L., Paolisso P., Belmonte M., Gallinoro E., Delrue L., Taktaz F., Fontanella R.A., Degrieck I., Pesapane A., Casselman F. (2024). Myocardial Sodium–Glucose Cotransporter 2 Expression and Cardiac Remodelling in Patients with Severe Aortic Stenosis: The BIO-AS Study. Eur. J. Heart Fail..

[99] Chung C.-C., Lin Y.-K., Chen Y.-C., Kao Y.-H., Yeh Y.-H., Trang N.N., Chen Y.-J. (2023). Empagliflozin Suppressed Cardiac Fibrogenesis through Sodium-Hydrogen Exchanger Inhibition and Modulation of the Calcium Homeostasis. Cardiovasc. Diabetol..

[100] Karakasis P., Patoulias D., Kassimis G., Koufakis T., Klisic A., Doumas M., Fragakis N., Rizzo M. (2024). Therapeutic Potential of Sodium-Glucose Co-Transporter-2 Inhibitors and Glucagon-like Peptide-1 Receptor Agonists for Patients with Acute Coronary Syndrome: A Review of Clinical Evidence. Curr. Pharm. Des..

[101] Packer M. (2022). Critical Reanalysis of the Mechanisms Underlying the Cardiorenal Benefits of SGLT2 Inhibitors and Reaffirmation of the Nutrient Deprivation Signaling/Autophagy Hypothesis. Circulation.

[102] Gager G.M., von Lewinski D., Sourij H., Jilma B., Eyileten C., Filipiak K., Hülsmann M., Kubica J., Postula M., Siller-Matula J.M. (2021). Effects of SGLT2 Inhibitors on Ion Homeostasis and Oxidative Stress Associated Mechanisms in Heart Failure. Biomed. Pharmacother..

[103] Verma S., Rawat S., Ho K.L., Wagg C.S., Zhang L., Teoh H., Dyck J.E., Uddin G.M., Oudit G.Y., Mayoux E. (2018). Empagliflozin Increases Cardiac Energy Production in Diabetes: Novel Translational Insights Into the Heart Failure Benefits of SGLT2 Inhibitors. JACC Basic Transl. Sci..

[104] Lu Q., Liu J., Li X., Sun X., Zhang J., Ren D., Tong N., Li J. (2020). Empagliflozin Attenuates Ischemia and Reperfusion Injury through LKB1/AMPK Signaling Pathway. Mol. Cell. Endocrinol..

[105] Chen M. (2023). Empagliflozin Attenuates Doxorubicin-Induced Cardiotoxicity by Activating AMPK/SIRT-1/PGC-1α-Mediated Mitochondrial Biogenesis. Toxicol. Res..

[106] Umino H., Hasegawa K., Minakuchi H., Muraoka H., Kawaguchi T., Kanda T., Tokuyama H., Wakino S., Itoh H. (2018). High Basolateral Glucose Increases Sodium-Glucose Cotransporter 2 and Reduces Sirtuin-1 in Renal Tubules through Glucose Transporter-2 Detection. Sci. Rep..

[107] Troise D., Mercuri S., Infante B., Losappio V., Cirolla L., Netti G.S., Ranieri E., Stallone G. (2024). MTOR and SGLT-2 Inhibitors: Their Synergistic Effect on Age-Related Processes. Int. J. Mol. Sci..

[108] Tomita I., Kume S., Sugahara S., Osawa N., Yamahara K., Yasuda-Yamahara M., Takeda N., Chin-Kanasaki M., Kaneko T., Mayoux E. (2020). SGLT2 Inhibition Mediates Protection from Diabetic Kidney Disease by Promoting Ketone Body-Induced MTORC1 Inhibition. Cell Metab..

[109] Jaikumkao K., Promsan S., Thongnak L., Swe M.T., Tapanya M., Htun K.T., Kothan S., Intachai N., Lungkaphin A. (2021). Dapagliflozin Ameliorates Pancreatic Injury and Activates Kidney Autophagy by Modulating the AMPK/MTOR Signaling Pathway in Obese Rats. J. Cell. Physiol..

[110] Lindman B.R., Dweck M.R., Lancellotti P., Généreux P., Piérard L.A., O’Gara P.T., Bonow R.O. (2020). Management of Asymptomatic Severe Aortic Stenosis: Evolving Concepts in Timing of Valve Replacement. JACC Cardiovasc. Imaging.

[111] Treibel T.A., Kozor R., Schofield R., Benedetti G., Fontana M., Bhuva A.N., Sheikh A., López B., González A., Manisty C. (2018). Reverse Myocardial Remodeling Following Valve Replacement in Patients with Aortic Stenosis. J. Am. Coll. Cardiol..

[112] Ducharme-Smith A., Chahal C.A.A., Sawatari H., Podboy A., Sherif A., Scott C.G., Brady P.A., Gersh B.J., Somers V.K., Nkomo V.T. (2020). Relationship Between Anemia and Sudden Cardiac Death in Patients with Severe Aortic Stenosis. Am. J. Cardiol..

[113] Jiménez-Xarrié E., Asmarats L., Roqué-Figuls M., Millán X., Li C.H.P., Fernández-Peregrina E., Sánchez-Ceña J., Massó van Roessel A., Maestre Hittinger M.L., Paniagua P. (2023). Impact of Baseline Anemia in Patients Undergoing Transcatheter Aortic Valve Replacement: A Prognostic Systematic Review and Meta-Analysis. J. Clin. Med..

[114] Mazer C.D., Hare G.M.T., Connelly P.W., Gilbert R.E., Shehata N., Quan A., Teoh H., Leiter L.A., Zinman B., Jüni P. (2020). Effect of Empagliflozin on Erythropoietin Levels, Iron Stores, and Red Blood Cell Morphology in Patients with Type 2 Diabetes Mellitus and Coronary Artery Disease. Circulation.

[115] Angermann C.E., Sehner S., Gerhardt L.M.S., Santos-Gallego C.G., Requena-Ibanez J.A., Zeller T., Maack C., Sanz J., Frantz S., Ertl G. (2025). Anaemia Predicts Iron Homoeostasis Dysregulation and Modulates the Response to Empagliflozin in Heart Failure with Reduced Ejection Fraction: The EMPATROPISM-FE Trial. Eur. Heart J..

[116] Packer M. (2023). Mechanisms of Enhanced Renal and Hepatic Erythropoietin Synthesis by Sodium–Glucose Cotransporter 2 Inhibitors. Eur. Heart J..

[117] Wang X., Fu R., Liu H., Ma Y., Qiu X., Dong Z. (2021). The Effects of Sodium Glucose Co-Transporter (SGLT) 2 Inhibitors on Hematocrit Levels: A Systematic Review and Meta-Analysis of Randomized Controlled Trials. Ann. Palliat. Med..

[118] Shah T., Zhang Z., Shah H., Fanaroff A.C., Nathan A.S., Parise H., Lutz J., Sugeng L., Bellumkonda L., Redfors B. (2025). Effect of Sodium-Glucose Cotransporter-2 Inhibitors on the Progression of Aortic Stenosis. JACC Cardiovasc. Interv..

[119] Jariwala P., Kulkarni G.P., Punjani A., Boorugu H., Gude D. (2024). Role of Empagliflozin in Heart Failure with Severe Aortic Stenosis before Valve Replacement: EASTER-HF Study. Indian Heart J..

[120] Paolisso P., Belmonte M., Gallinoro E., Scarsini R., Bergamaschi L., Portolan L., Armillotta M., Esposito G., Moscarella E., Benfari G. (2024). SGLT2-Inhibitors in Diabetic Patients with Severe Aortic Stenosis and Cardiac Damage Undergoing Transcatheter Aortic Valve Implantation (TAVI). Cardiovasc. Diabetol..

[121] Raposeiras-Roubín S., Amat-Santos I.J., Rossello X., González Ferreiro R., González Bermúdez I., Lopez Otero D., Nombela-Franco L., Gheorghe L., Diez J.L., Baladrón Zorita C. (2025). Dapagliflozin in Patients Undergoing Transcatheter Aortic-Valve Implantation. N. Engl. J. Med..

[122] Thakkar S., Nair A., Samimi S., Kharsa C., Zaid S., Aoun J., Desai P., Faza N., Little S., Atkins M. (2024). TCT-184 Comparative Outcomes of SGLT2 Inhibitors in Patients Undergoing Transcatheter Aortic Valve Replacement. J. Am. Coll. Cardiol..

[123] Abdelfattah O.M., Jacquemyn X., Sá M.P., Jneid H., Sultan I., Cohen D.J., Gillam L.D., Aron L., Clavel M.-A., Pibarot P. (2024). Cardiac Damage Staging Predicts Outcomes in Aortic Valve Stenosis After Aortic Valve Replacement: Meta-Analysis. JACC Adv..

[124] Okumus N., Abraham S., Puri R., Tang W.H.W. (2023). Aortic Valve Disease, Transcatheter Aortic Valve Replacement, and the Heart Failure Patient: A State-of-the-Art Review. JACC Heart Fail..

[125] Thornton G.D., Musa T.A., Rigolli M., Loudon M., Chin C., Pica S., Malley T., Foley J.R.J., Vassiliou V.S., Davies R.H. (2022). Association of Myocardial Fibrosis and Stroke Volume by Cardiovascular Magnetic Resonance in Patients with Severe Aortic Stenosis with Outcome After Valve Replacement: The British Society of Cardiovascular Magnetic Resonance AS700 Study. JAMA Cardiol..

[126] Evertz R., Hub S., Beuthner B.E., Backhaus S.J., Lange T., Schulz A., Toischer K., Seidler T., von Haehling S., Puls M. (2023). Aortic Valve Calcification and Myocardial Fibrosis Determine Outcome Following Transcatheter Aortic Valve Replacement. ESC Heart Fail..

[127] Fukutomi M., Onishi T., Ando T., Higuchi R., Hagiya K., Saji M., Takamisawa I., Iguchi N., Takayama M., Shimizu A. (2024). Impact of Prior Hospitalization for Heart Failure on Clinical Outcomes of Patients after Transcatheter Aortic Valve Implantation with New-Generation Devices: Insights from the LAPLACE-TAVI Registry. Catheter. Cardiovasc. Interv..

[128] Puls M., Beuthner B.E., Topci R., Jacob C.F., Steinhaus K.E., Paul N., Beißbarth T., Toischer K., Jacobshagen C., Hasenfuß G. (2024). Patients with Paradoxical Low-Flow, Low-Gradient Aortic Stenosis Gain the Least Benefit from TAVI among All Hemodynamic Subtypes. Clin. Res. Cardiol..

[129] Thyregod H.G.H., Jørgensen T.H., Ihlemann N., Steinbrüchel D.A., Nissen H., Kjeldsen B.J., Petursson P., De Backer O., Olsen P.S., Søndergaard L. (2024). Transcatheter or Surgical Aortic Valve Implantation: 10-Year Outcomes of the NOTION Trial. Eur. Heart J..

[130] Lopez-Martinez H., Vilalta V., Farjat-Pasos J., Ferrer-Sistach E., Mohammadi S., Escabia C., Kalavrouziotis D., Resta H., Borrellas A., Dumont E. (2024). Heart Failure Hospitalization Following Surgical or Transcatheter Aortic Valve Implantation in Low-Risk Aortic Stenosis. ESC Heart Fail..

[131] Saijo Y., Kusunose K., Takahashi T., Yamada H., Sata M., Sato K., Albakaa N., Ishizu T., Seo Y., Izumo M. (2024). Impact of Transcatheter Aortic Valve Replacement on Cardiac Reverse Remodeling and Prognosis in Mixed Aortic Valve Disease. J. Am. Heart Assoc..

[132] Wagener M., Reuthebuch O., Heg D., Tüller D., Ferrari E., Grünenfelder J., Huber C., Moarof I., Muller O., Nietlispach F. (2023). Clinical Outcomes in High-Gradient, Classical Low-Flow, Low-Gradient, and Paradoxical Low-Flow, Low-Gradient Aortic Stenosis After Transcatheter Aortic Valve Implantation: A Report from the SwissTAVI Registry. J. Am. Heart Assoc..

[133] Jakulla R.S., Gunta S.P., Huded C.P. (2023). Heart Failure after Aortic Valve Replacement: Incidence, Risk Factors, and Implications. J. Clin. Med..

[134] Amat-Santos I.J., Sánchez-Luna J.P., Abu-Assi E., Melendo-Viu M., Cruz-Gonzalez I., Nombela-Franco L., Muñoz-Garcí A.J., Blas S.G., de la Torre Hernandez J.M., Romaguera R. (2022). Rationale and Design of the Dapagliflozin after Transcatheter Aortic Valve Implantation (DapaTAVI) Randomized Trial. Eur. J. Heart Fail..

[135] Usman M.S., Bhatt D.L., Hameed I., Anker S.D., Cheng A.Y.Y., Hernandez A.F., Jones W.S., Khan M.S., Petrie M.C., Udell J.A. (2024). Effect of SGLT2 Inhibitors on Heart Failure Outcomes and Cardiovascular Death across the Cardiometabolic Disease Spectrum: A Systematic Review and Meta-Analysis. Lancet Diabetes Endocrinol..

[136] Usman M.S., Siddiqi T.J., Anker S.D., Bakris G.L., Bhatt D.L., Filippatos G., Fonarow G.C., Greene S.J., Januzzi J.L.J., Khan M.S. (2023). Effect of SGLT2 Inhibitors on Cardiovascular Outcomes Across Various Patient Populations. J. Am. Coll. Cardiol..

[137] Karakasis P., Pamporis K., Stachteas P., Patoulias D., Bougioukas K.I., Fragakis N. (2023). Efficacy and Safety of Sodium-Glucose Cotransporter-2 Inhibitors in Heart Failure with Mildly Reduced or Preserved Ejection Fraction: An Overview of 36 Systematic Reviews. Heart Fail. Rev..

[138] Stachteas P., Nasoufidou A., Patoulias D., Karakasis P., Karagiannidis E., Mourtzos M.-A., Samaras A., Apostolidou X., Fragakis N. (2024). The Role of Sodium-Glucose Co-Transporter-2 Inhibitors on Diuretic Resistance in Heart Failure. Int. J. Mol. Sci..

[139] Karakasis P., Theofilis P., Patoulias D., Schuermans A., Vlachakis P.K., Klisic A., Rizzo M., Fragakis N. (2025). Sodium-Glucose Cotransporter 2 Inhibitors and Outcomes in Transthyretin Amyloid Cardiomyopathy: Systematic Review and Meta-Analysis. Eur. J. Clin. Investig..

[140] Karakasis P., Patoulias D., Giannakoulas G., Rosenkranz S., Fragakis N. (2024). Effect of Sodium-Glucose Cotransporter-2 Inhibitors on Pulmonary Arterial Wedge Pressure. Eur. J. Intern. Med..

[141] Olivotto I., Oreziak A., Barriales-Villa R., Abraham T.P., Masri A., Garcia-Pavia P., Saberi S., Lakdawala N.K., Wheeler M.T., Owens A. (2020). Mavacamten for Treatment of Symptomatic Obstructive Hypertrophic Cardiomyopathy (EXPLORER-HCM): A Randomised, Double-Blind, Placebo-Controlled, Phase 3 Trial. Lancet.

[142] Ho C.Y., Mealiffe M.E., Bach R.G., Bhattacharya M., Choudhury L., Edelberg J.M., Hegde S.M., Jacoby D., Lakdawala N.K., Lester S.J. (2020). Evaluation of Mavacamten in Symptomatic Patients with Nonobstructive Hypertrophic Cardiomyopathy. J. Am. Coll. Cardiol..

[143] Lim G.B. (2021). Drugs Targeting the Sarcomere in Heart Failure and Hypertrophic Cardiomyopathy. Nat. Rev. Cardiol..

[144] Saberi S., Cardim N., Yamani M., Schulz-Menger J., Li W., Florea V., Sehnert A.J., Kwong R.Y., Jerosch-Herold M., Masri A. (2021). Mavacamten Favorably Impacts Cardiac Structure in Obstructive Hypertrophic Cardiomyopathy: EXPLORER-HCM Cardiac Magnetic Resonance Substudy Analysis. Circulation.

[145] Hansson N.H., Sörensen J., Harms H.J., Kim W.Y., Nielsen R., Tolbod L.P., Frøkiær J., Bouchelouche K., Dodt K.K., Sihm I. (2017). Metoprolol Reduces Hemodynamic and Metabolic Overload in Asymptomatic Aortic Valve Stenosis Patients: A Randomized Trial. Circ. Cardiovasc. Imaging.

[146] Rossi A., Temporelli P.L., Cicoira M., Gaibazzi N., Cioffi G., Nistri S., Magatelli M., Tavazzi L., Faggiano P. (2015). Beta-Blockers Can Improve Survival in Medically-Treated Patients with Severe Symptomatic Aortic Stenosis. Int. J. Cardiol..

[147] Hansson E.C., Martinsson A., Baranowska J., Törngren C., Pan E., Björklund E., Karlsson M. (2024). Betablockers and Clinical Outcome after Surgical Aortic Valve Replacement: A Report from the SWEDEHEART Registry. Eur. J. Cardio-Thorac. Surg..

[148] Bang C.N., Greve A.M., Rossebø A.B., Ray S., Egstrup K., Boman K., Nienaber C., Okin P.M., Devereux R.B., Wachtell K. (2017). Antihypertensive Treatment with β-Blockade in Patients with Asymptomatic Aortic Stenosis and Association with Cardiovascular Events. J. Am. Heart Assoc..

[149] Shumkova M., De Oliveira E.O., Hrubyak L.H., Beles M.B., Bertolone D.T.B., Ratti A.R., Adeo L.A., Viscusi M.V., Barbato E.B., Wyffels E.W. (2024). Continuation of Beta-Blockers at Discharge Is Associated with Improved Outcome in Patients with HFpEF and Concomitant Moderate Aortic Stenosis. Eur. Heart J..

[150] Hopfgarten J., James S., Lindhagen L., Baron T., Ståhle E., Christersson C. (2024). Medical Treatment of Heart Failure with Renin-Angiotensin-Aldosterone System Inhibitors and Beta-Blockers in Aortic Stenosis: Association with Long-Term Outcome after Aortic Valve Replacement. Eur. Heart J. Open.

[151] Bull S., Loudon M., Francis J.M., Joseph J., Gerry S., Karamitsos T.D., Prendergast B.D., Banning A.P., Neubauer S., Myerson S.G. (2015). A Prospective, Double-Blind, Randomized Controlled Trial of the Angiotensin-Converting Enzyme Inhibitor Ramipril in Aortic Stenosis (RIAS Trial). Eur. Heart J. Cardiovasc. Imaging.

[152] Goel S.S., Kleiman N.S., Zoghbi W.A., Reardon M.J., Kapadia S.R. (2020). Renin-Angiotensin System Blockade in Aortic Stenosis: Implications Before and After Aortic Valve Replacement. J. Am. Heart Assoc..

[153] Ochiai T., Saito S., Yamanaka F., Shishido K., Tanaka Y., Yamabe T., Shirai S., Tada N., Araki M., Naganuma T. (2018). Renin-Angiotensin System Blockade Therapy after Transcatheter Aortic Valve Implantation. Heart.

[154] Rodriguez-Gabella T., Catalá P., Muñoz-García A.J., Nombela-Franco L., Del Valle R., Gutiérrez E., Regueiro A., Jimenez-Diaz V.A., Ribeiro H.B., Rivero F. (2019). Renin-Angiotensin System Inhibition Following Transcatheter Aortic Valve Replacement. J. Am. Coll. Cardiol..

[155] Inohara T., Manandhar P., Kosinski A.S., Matsouaka R.A., Kohsaka S., Mentz R.J., Thourani V.H., Carroll J.D., Kirtane A.J., Bavaria J.E. (2018). Association of Renin-Angiotensin Inhibitor Treatment with Mortality and Heart Failure Readmission in Patients with Transcatheter Aortic Valve Replacement. JAMA.

[156] Chen S., Redfors B., Nazif T., Kirtane A., Crowley A., Ben-Yehuda O., Kapadia S., Finn M.T., Goel S., Lindman B.R. (2020). Impact of Renin–Angiotensin System Inhibitors on Clinical Outcomes in Patients with Severe Aortic Stenosis Undergoing Transcatheter Aortic Valve Replacement: An Analysis of from the PARTNER 2 Trial and Registries. Eur. Heart J..

[157] Chockalingam A., Venkatesan S., Subramaniam T., Jagannathan V., Elangovan S., Alagesan R., Gnanavelu G., Dorairajan S., Krishna B.P., Chockalingam V. (2004). Safety and Efficacy of Angiotensin-Converting Enzyme Inhibitors in Symptomatic Severe Aortic Stenosis: Symptomatic Cardiac Obstruction-Pilot Study of Enalapril in Aortic Stenosis (SCOPE-AS). Am. Heart J..

[158] Bang C.N., Greve A.M., Køber L., Rossebø A.B., Ray S., Boman K., Nienaber C.A., Devereux R.B., Wachtell K. (2014). Renin-Angiotensin System Inhibition Is Not Associated with Increased Sudden Cardiac Death, Cardiovascular Mortality or All-Cause Mortality in Patients with Aortic Stenosis. Int. J. Cardiol..

[159] Dalsgaard M., Iversen K., Kjaergaard J., Grande P., Goetze J.P., Clemmensen P., Hassager C. (2014). Short-Term Hemodynamic Effect of Angiotensin-Converting Enzyme Inhibition in Patients with Severe Aortic Stenosis: A Placebo-Controlled, Randomized Study. Am. Heart J..

[160] Yamamoto K., Takeji Y., Taniguchi T., Morimoto T., Shirai S., Kitai T., Tabata H., Ohno N., Murai R., Osakada K. (2024). Safety of Calcium Channel Blockers in Patients with Severe Aortic Stenosis and Hypertension. Circ. J..

[161] Saeed S., Mancia G., Rajani R., Parkin D., Chambers J.B. (2020). Antihypertensive Treatment with Calcium Channel Blockers in Patients with Moderate or Severe Aortic Stenosis: Relationship with All-Cause Mortality. Int. J. Cardiol..

[162] Miyahara D., Izumo M., Sato Y., Shoji T., Yamaga M., Sekiguchi M., Tanaka T., Kobayashi Y., Kai T., Okuno T. (2025). Calcium Channel Blocker Use and Outcomes Following Transcatheter Aortic Valve Intervention for Aortic Stenosis. Cardiovasc. Interv. Ther..

[163] Claveau D., Piha-Gossack A., Friedland S.N., Afilalo J., Rudski L. (2015). Complications Associated with Nitrate Use in Patients Presenting with Acute Pulmonary Edema and Concomitant Moderate or Severe Aortic Stenosis. Ann. Emerg. Med..

[164] Costa G., Silva A.L., Ramalho R., Vieira M.J., Goncalves L., Teixeira R. (2024). The Use of Sublingual Nitroglycerin in Patients with Severe Aortic Stenosis before Cardiac CT-Prospective Suty. Eur. Hear. J.-Cardiovasc. Imaging.

[165] Vahanian A., Beyersdorf F., Praz F., Milojevic M., Baldus S., Bauersachs J., Capodanno D., Conradi L., De Bonis M., De Paulis R. (2022). 2021 ESC/EACTS Guidelines for the Management of Valvular Heart Disease. Eur. Heart J..

[166] McDonagh T.A., Metra M., Adamo M., Gardner R.S., Baumbach A., Böhm M., Burri H., Butler J., Čelutkienė J., Chioncel O. (2023). 2023 Focused Update of the 2021 ESC Guidelines for the Diagnosis and Treatment of Acute and Chronic Heart Failure. Eur. Heart J..

