# Supplementary material for: Sodium–Glucose Cotransporter 2 Inhibitors in Aortic Stenosis: Toward a Comprehensive Cardiometabolic Approach
Source: Int J Mol Sci. 2025 May 8;26(10):4494. doi: 10.3390/ijms26104494 (PMC12111810; doi:10.3390/ijms26104494)
Supplement: Supplementary file 1 [file ijms-26-04494-s001.zip › ijms-3627153-supplementary.pdf]

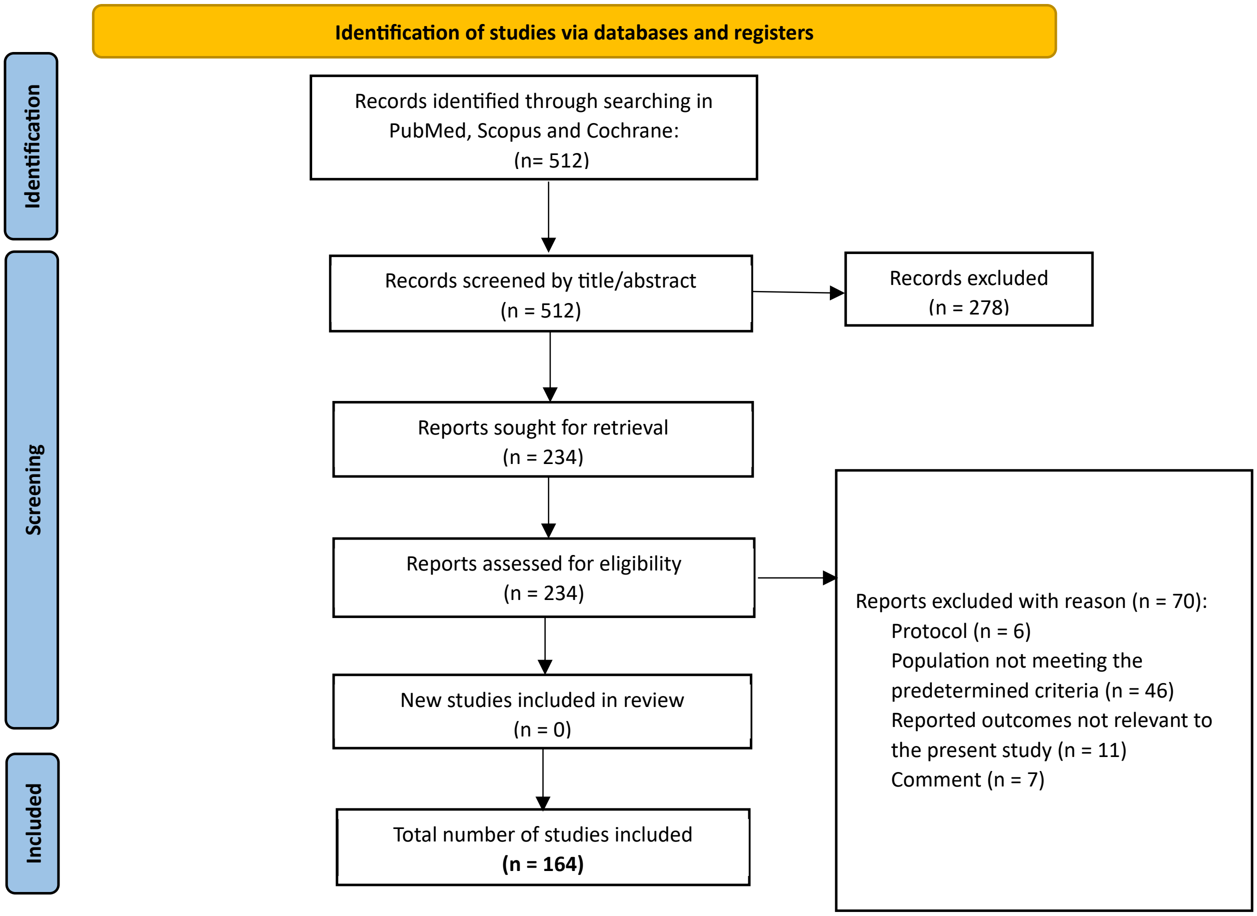


**Supplementary Figure S1.** Preferred Reporting Items for Systematic Reviews and Meta-Analyses (PRISMA) flow diagram.
